# Tumour-selective activity of RAS-GTP inhibition in pancreatic cancer

**DOI:** 10.1038/s41586-024-07379-z

**Published:** 2024-04-08

**Authors:** Urszula N. Wasko, Jingjing Jiang, Tanner C. Dalton, Alvaro Curiel-Garcia, A. Cole Edwards, Yingyun Wang, Bianca Lee, Margo Orlen, Sha Tian, Clint A. Stalnecker, Kristina Drizyte-Miller, Marie Menard, Julien Dilly, Stephen A. Sastra, Carmine F. Palermo, Marie C. Hasselluhn, Amanda R. Decker-Farrell, Stephanie Chang, Lingyan Jiang, Xing Wei, Yu C. Yang, Ciara Helland, Haley Courtney, Yevgeniy Gindin, Karl Muonio, Ruiping Zhao, Samantha B. Kemp, Cynthia Clendenin, Rina Sor, William P. Vostrejs, Priya S. Hibshman, Amber M. Amparo, Connor Hennessey, Matthew G. Rees, Melissa M. Ronan, Jennifer A. Roth, Jens Brodbeck, Lorenzo Tomassoni, Basil Bakir, Nicholas D. Socci, Laura E. Herring, Natalie K. Barker, Junning Wang, James M. Cleary, Brian M. Wolpin, John A. Chabot, Michael D. Kluger, Gulam A. Manji, Kenneth Y. Tsai, Miroslav Sekulic, Stephen M. Lagana, Andrea Califano, Elsa Quintana, Zhengping Wang, Jacqueline A. M. Smith, Matthew Holderfield, David Wildes, Scott W. Lowe, Michael A. Badgley, Andrew J. Aguirre, Robert H. Vonderheide, Ben Z. Stanger, Timour Baslan, Channing J. Der, Mallika Singh, Kenneth P. Olive

**Affiliations:** 1 Department of Medicine, Vagelos College of Physicians and Surgeons, Columbia University Irving Medical Center, New York, NY USA; 2 Herbert Irving Comprehensive Cancer Center, Columbia University Irving Medical Center, New York, NY USA; 3 Revolution Medicines, Redwood City, CA USA; 4 Department of Cell Biology and Physiology, University of North Carolina at Chapel Hill, Chapel Hill, NC USA; 5 University of Pennsylvania Perelman School of Medicine, Department of Medicine, Philadelphia, PA USA; 6 Cancer Biology and Genetics Program, Sloan Kettering Institute, Memorial Sloan Kettering Cancer Center, New York, NY USA; 7 Lineberger Comprehensive Cancer Center, University of North Carolina at Chapel Hill, Chapel Hill, NC USA; 8 Department of Pharmacology, University of North Carolina at Chapel Hill, Chapel Hill, NC USA; 9 Department of Medical Oncology, Dana-Farber Cancer Institute, Boston, MA USA; 10 Harvard Medical School, Boston, MA USA; 11 University of Pennsylvania Perelman School of Medicine, Abramson Cancer Center, Philadelphia, PA USA; 12 The Broad Institute of Harvard and MIT, Cambridge, MA USA; 13 Department of Systems Biology, Columbia University Irving Medical Center, New York, NY USA; 14 Bioinformatics Core, Memorial Sloan Kettering Cancer Center, New York, NY USA; 15 UNC Michael Hooker Proteomics Center, University of North Carolina at Chapel Hill, Chapel Hill, NC USA; 16 Department of Surgery, Vagelos College of Physicians and Surgeons, Columbia University Irving Medical Center, New York, NY USA; 17 Department of Pathology, H. Lee Moffitt Cancer Center and Research Institute, Tampa, FL USA; 18 Department of Tumor Microenvironment and Metastasis, H. Lee Moffitt Cancer Center and Research Institute, Tampa, FL USA; 19 Department of Pathology and Cell Biology, Columbia University Irving Medical Center, New York, NY USA; 20 Department of Oncology, The Johns Hopkins University School of Medicine, Baltimore, MD USA; 21 J. P. Sulzberger Columbia Genome Center, Columbia University, New York, NY USA; 22 Department of Biochemistry and Molecular Biophysics, Columbia University Irving Medical Center, New York, NY USA; 23 Department of Biomedical Informatics, Columbia University Irving Medical Center, New York, NY USA; 24 Chan Zuckerberg Biohub New York, New York, NY USA; 25 Howard Hughes Medical Institute, Memorial Sloan Kettering Cancer Center, New York, NY USA; 26 Department of Medicine, Brigham and Women’s Hospital, Boston, MA USA; 27 Parker Institute for Cancer Immunotherapy, San Francisco, CA USA; 28 Department of Biomedical Sciences, School of Veterinary Medicine, The University of Pennsylvania, Philadelphia, PA USA

**Keywords:** Pancreatic cancer, Pharmacodynamics

## Abstract

Broad-spectrum RAS inhibition has the potential to benefit roughly a quarter of human patients with cancer whose tumours are driven by RAS mutations^1,2^. RMC-7977 is a highly selective inhibitor of the active GTP-bound forms of KRAS, HRAS and NRAS, with affinity for both mutant and wild-type variants^3^. More than 90% of cases of human pancreatic ductal adenocarcinoma (PDAC) are driven by activating mutations in KRAS^4^. Here we assessed the therapeutic potential of RMC-7977 in a comprehensive range of PDAC models. We observed broad and pronounced anti-tumour activity across models following direct RAS inhibition at exposures that were well-tolerated in vivo. Pharmacological analyses revealed divergent responses to RMC-7977 in tumour versus normal tissues. Treated tumours exhibited waves of apoptosis along with sustained proliferative arrest, whereas normal tissues underwent only transient decreases in proliferation, with no evidence of apoptosis. In the autochthonous KPC mouse model, RMC-7977 treatment resulted in a profound extension of survival followed by on-treatment relapse. Analysis of relapsed tumours identified *Myc* copy number gain as a prevalent candidate resistance mechanism, which could be overcome by combinatorial TEAD inhibition in vitro. Together, these data establish a strong preclinical rationale for the use of broad-spectrum RAS-GTP inhibition in the setting of PDAC and identify a promising candidate combination therapeutic regimen to overcome monotherapy resistance.

## Abstract (web summary)

RMC-7977, a multi-selective RAS(ON) inhibitor, exhibits potent tumour-selective activity in multiple pre-clinical models of pancreatic ductal adenocarcinoma through a combination of pharmacology and oncogene dependence.

## Main

Activating mutations in the three isoforms of the RAS oncogene (*HRAS*, *KRAS* and *NRAS*) are associated with around 20–30% of human cancers^1,2^. *KRAS* is the predominant isoform that is mutated in cancer, including in more than 90% of cases of PDAC, a leading cause of cancer mortality in the USA^4^ and globally. Although RAS proteins were long regarded as undruggable, recent advances have led to the development and approval of agents that target one specific RAS variant, KRAS(G12C)^5,6^. This strategy has shown promising efficacy in *KRAS*^G12C^-mutant tumours, including the small fraction (around 1%) of PDAC cases with this allele^7,8^. However, resistance arises quickly in the majority of patients treated with KRAS(G12C) inhibitors and various alterations that reactivate RAS signalling both directly and indirectly have been identified in patients who have progressed on these inhibitors^9–11^. The KRAS(G12C) inhibitors that are approved by the US Food and Drug Administration, as well as two recently described inhibitors (one that targets KRAS(G12D) and one with broader specificity for KRAS mutants), selectively target the inactive, GDP-bound state of mutant KRAS, and are consequently vulnerable to mechanisms of resistance that increase levels of GTP-bound KRAS or wild-type HRAS and NRAS, including activation of upstream receptor tyrosine kinases^12–14^. Here we used the tool compound RMC-7977 to evaluate the pharmacology and anti-tumour activity of multi-selective inhibitors with activity for the active state of RAS family proteins (RAS(ON) inhibitors) in preclinical models of PDAC. This mechanistically distinct class of RAS(ON) multi-selective tri-complex inhibitors, which includes the investigational agent RMC-6236, exhibits selectivity for the active, GTP-bound forms of all RAS isoforms, both mutant and wild type. In the accompanying Article, Singh et al.^3^ describe the identification of RMC-7977 along with evidence that this agent can overcome some forms of acquired resistance to inhibitors that target GDP-bound RAS isoforms. RMC-6236 is currently in early clinical evaluation in patients with advanced solid tumours with RAS mutations (ClinicalTrials.gov identifier: NCT05379985).

The singular role of mutant KRAS in PDAC oncogenesis has inspired several strategies for therapeutic intervention. Efforts to target prenylation of RAS proteins, upstream receptors and downstream signalling have been hampered by functional redundancies and compensatory feedback mechanisms^1,3,10,11,15,16^. Combinatorial strategies that target multiple pathway effectors or compensatory responses can drive greater activity, but this is generally at the cost of reduced tolerability. Studies of *Kras*^G12D^ gene deletion in engineered mouse models have demonstrated that mutant KRAS has an essential role in the maintenance of PDAC^17–19^. This was recently supported by preclinical evidence showing tumour regressions in PDAC models^12,13^ following pharmacologic inhibition of KRAS(G12D). The development of RAS inhibitors with broader specificity has the potential to benefit the majority of patients with PDAC while countering a wider range of resistance mechanisms. However, given the critical role of RAS proteins in normal tissue homeostasis^20,21^, a prevailing question is whether broad inhibition of RAS activity in tumours can be implemented with a suitable therapeutic index^22^.

## RMC-7977 is active in PDAC models

**Fig. 1:**
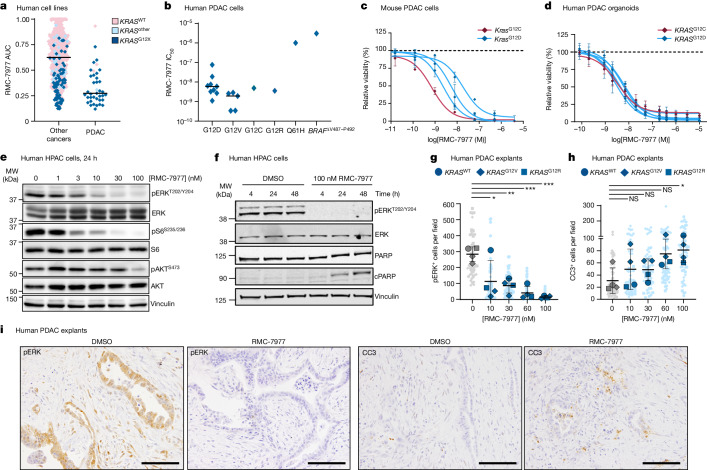
RMC-7977 exhibits potent anti-tumour activity in in vitro models of PDAC. **a**, PRISM multiplex screening changes in viability of 796 cancer cell lines in response to RMC-7977 treatment. Cell line viability was plotted as area under the curve (AUC) values. Colours indicate *KRAS* status. Horizontal lines indicate median. WT, wild type. **b**, Sensitivity of human PDAC cell lines with *KRAS*^G12X^, *KRAS*^Q61H^ or *BRAF*^ΔV487–P492^ mutations treated with indicated concentrations of RMC-7977 for 5 days, expressed as half-maximal inhibitory concentration (IC_50_). **c**, Viability of mouse PDAC lines with *Kras*^G12X^ mutations treated with indicated concentrations of RMC-7977 for 72 h. **d**, Viability of human PDAC organoids with *KRAS*^G12X^ mutations treated with indicated concentrations of RMC-7977 for 6 days. Data in **b**–**d** are mean ± s.d. of three biological replicates normalized to DMSO control. Colours indicate *KRAS* mutation. **e**, Western blots of HPAC cells treated with DMSO or RMC-7977 at the indicated concentrations for 24 h (*n* = 3). MW, molecular weight. **f**, Western blots of HPAC cells treated with DMSO or 100 nM RMC-7977 for indicated durations. **e**,**f**, Vinculin was used as loading control. **g**–**i**, Ex vivo human PDAC explants treated with DMSO or indicated concentrations of RMC-7977 for 24 h (*n* = 4). **g**,**h**, Quantification of pERK^T202/Y204^ (**g**) and CC3 (**h**) from IHC analysis of explants. Analysis based on 10–15 fields of view (light shade), averaged per explant slice (dark shade) compared by one-way analysis of variance (ANOVA) with Tukey correction. Error bars indicate ±s.d. **i**, Representative IHC of pERK^T202/Y204^ and CC3 staining of explants treated with DMSO or 100 nM RMC-7977. Scale bars, 50 μm. Cell line information is provided in Supplementary Table 5. *P < 0.05, **P < 0.01, ***P < 0.001, ****P < 0.0001; NS, not significant. Source Data

**Extended Data Fig. 1:**
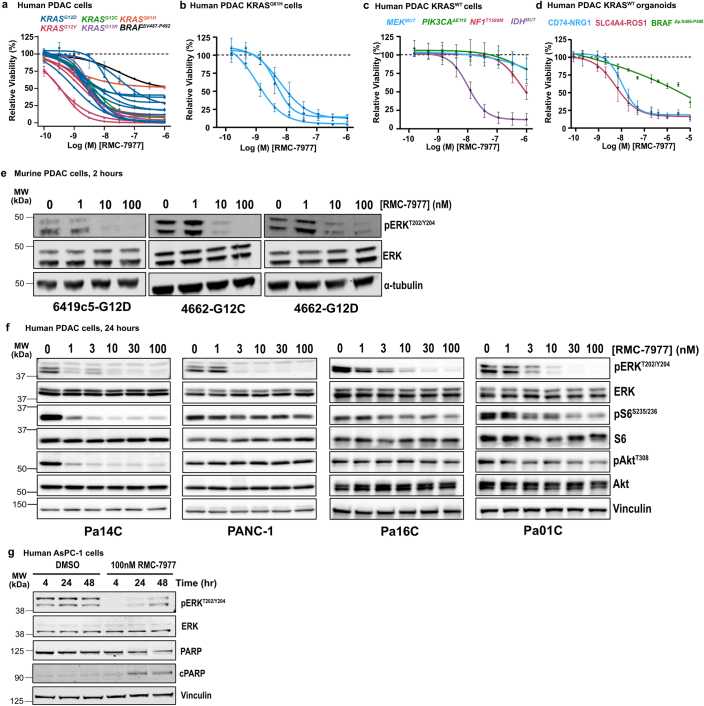
**(a)** Viability levels of human PDAC cell lines (from Fig. 1b) with *KRAS*^G12X^, *KRAS*^Q61H^, and *BRAF*^ΔV487-P492^ mutations treated with indicated concentrations of RMC-7977 for 5 days. Data points represent the mean of biological 3 replicates normalized to DMSO control. *KRAS* mutations are indicated by curve colors. Error bars indicate ±s.d. **(b,c)** Viability levels of human PDAC cell lines with **(b)** *KRAS*^Q61H^ mutation or **(c)** *KRAS*^WT^ treated with DMSO or indicated concentrations of RMC-7977 for 5 days. Data points represent the mean of 3-4 biological replicates normalized to DMSO control. Error bars indicate ±s.d. **(d)** Viability levels of human PDAC organoids with *KRAS*^WT^ treated with DMSO or indicated concentrations of RMC-7977 for 6 days. Data points represent the mean of 2 biological replicates normalized to DMSO control. Error bars indicate ±s.d. **(e)** Western Blots of mouse PDAC cell lines treated with DMSO or range of RMC-7977 concentrations (1-100 nM) for 2 h. Protein levels of phospho-ERK^T202/Y204^ and total ERK were analyzed. Alpha-(α)-tubulin was used as loading control. **(f)** Western Blots of human PDAC cell lines treated with DMSO or range of RMC-7977 concentrations (1-100 nM) for 24 h. Protein levels of phospho-ERK^T202/Y204^, total ERK, phospho-pS6^S235/236^ total S6, phospho-Akt^T308^ and total Akt were analyzed. Vinculin was used as loading control. **(g)** Western Blots of AsPC-1 cell line treated with DMSO or RMC-7977 (100 nM) for indicated timepoints. Protein levels of phospho-ERK^T202/Y204^, total ERK, total PARP and cleaved PARP were analyzed. Vinculin was used as loading control. For cell line information see SI Table 5. Source Data

*KRAS* mutations^23^ in PDAC occur principally at codon 12 (*KRAS*^G12X^) with infrequent occurrence of mutations at codons 61 (6–7%) and 13 (1%). Consistent with the finding that cell lines with *KRAS*^G12X^ mutations are particularly sensitive to RMC-7977^3^, we found that human PDAC cell lines were among the most sensitive in a large-scale screen of 796 human tumour cell lines using the PRISM platform (Fig. 1a). RMC-7977 exhibited low nanomolar potency on the viability of most human and mouse PDAC cell lines and human PDAC organoids (Fig. 1b–d). Two human cell lines showed lower sensitivity in vitro, one with *KRAS*^Q61H^ and one with wild-type *KRAS*; the cell line with wild-type *KRAS* had a class II *BRAF* mutation, which was predicted to be independent of RAS-GTP inhibition (Fig. 1b). Analysis of three additional *KRAS*^Q61H^ PDAC lines found low nanomolar potency of RMC-7977 for each (Extended data Fig. 1b) and an examination of *KRAS* wild-type cell lines and organoids indicated a wider range of sensitivity that is likely to reflect the nature of the respective driving mutations (Extended Data Fig. 1c,d). Western blot analyses of human and mouse PDAC cell lines showed reduced phosphorylation of the RAS–RAF effector proteins ERK1/2, indicating effective inhibition of the RAS–MAPK pathway at concentrations consistent with observed half-maximal growth inhibition (GI_50_) values (Fig. 1e and Extended Data Fig. 1e,f). In human cell lines, we detected more variable inhibition of PI3K effector signalling, as monitored by phosphorylation of AKT (pAKT) and S6 (pS6^S235/S236^), indicating some heterogeneity in the signalling responses across different lines and consistent with additional inputs (beyond direct RAS interactions) driving PI3K signalling in some contexts (Fig. 1e and Extended Data Fig. 1e). Inhibition of ERK1/2 phosphorylation (pERK) was generally durable over 48 h in human cell lines and associated with induction of the apoptotic marker cleaved PARP (cPARP) at later timepoints (Fig. 1f and Extended Data Fig. 1f).

Multiple studies have demonstrated that stromal cells resident in PDAC tissues can modulate the sensitivity of malignant cells to therapy^24^. To assess the potency of RMC-7977 in the context of an intact, all-human microenvironment, we used an ex vivo human PDAC explant model^25^, comprising cultured intact slices of freshly resected human PDAC tissue from patients at New York Presbyterian Hospital and Columbia University Irving Medical Center. Immunohistochemistry (IHC) performed on PDAC explants treated for 24 h with RMC-7977 showed a concentration-dependent decrease in pERK expression with an accompanying increase in the apoptosis marker cleaved caspase-3 (CC3), with maximal changes at 100 nM, the highest concentration tested (Fig. 1g–i). Of note, one of the four explant models was found to be wild type for KRAS and was also sensitive to RMC-7977 (Fig. 1g, circles). Together, these data are consistent with the pharmacodynamic responses observed in isolated cell lines and 3D organoid systems and imply that the consequences of direct RAS inhibition observed in vitro are recapitulated in a complex PDAC tumour milieu. In summary, RMC-7977 consistently and potently inhibited RAS pathway signalling across PDAC cell lines, patient-derived organoids and human tumour explants, resulting in growth attenuation and/or induction of apoptosis.

**Fig. 2:**
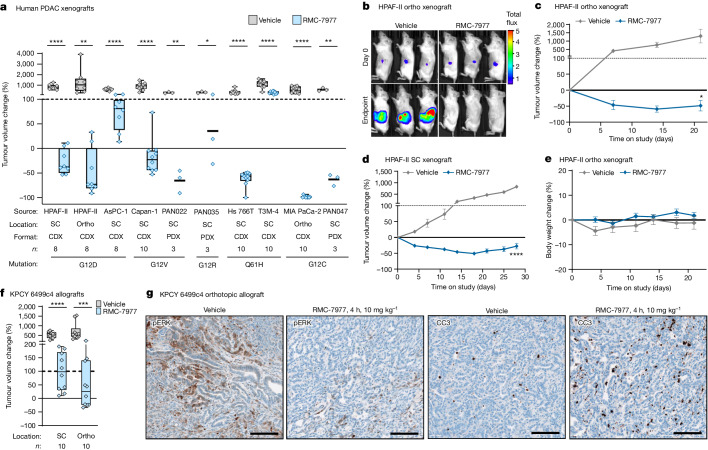
RMC-7977 exhibits anti-tumour activity in xenograft and allograft models of PDAC. **a**–**e**, Human PDAC xenograft models implanted either subcutaneously (SC) or orthotopically (ortho) into immunodeficient mice. Tumour-bearing mice were treated with vehicle or 10 mg kg^−1^ RMC-7977 orally, once daily, for 21–28 days. **a**, Box plot showing percentage change in tumour volume at endpoint compared with baseline at day 0 in vehicle and RMC-7977 treatment arms. Each symbol represents one mouse. Source and format of cell line, *KRAS* mutation, number of mice and tumour location are indicated. Study arms were compared by two-tailed Student’s unpaired *t*-test. **b**, Representative bioluminescence images showing signal in HPAF-II orthotropic xenograft tumours at day 0 and day 21 (endpoint) for vehicle and RMC-7977 treatment arms. **c**,**d**, Representative tumour growth curves for HPAF-II orthotopic (**c**) and subcutaneous (**d**) xenograft models treated with vehicle or RMC-7977, shown as percentage change in tumour volume from baseline over time. Vehicle and RMC-7977 groups were compared by two-way repeated measures ANOVA on the last measurement day of the vehicle group. Data are mean ± s.e.m. **e**, Tolerability of RMC-7977 as assessed by percentage change in body weight from baseline over time. Data are mean ± s.e.m. **f**,**g**, The KPCY-derived PDAC cell line 6499c4 was transplanted either subcutaneously or orthotopically into syngeneic mice. Tumour-bearing mice were treated with vehicle or 10 mg kg^−1^ RMC-7977 orally, once daily. **f**, Box plot showing changes in tumour volumes at day 14, compared with baseline at day 0, in vehicle and RMC-7977 treatment arms. Groups compared by two-tailed unpaired Student’s *t*-test. Tumour locations as indicated in the graph. **g**, Representative IHC of pERK^T202/Y204^ (left) or CC3 (right) in tumours from vehicle and RMC-7977-treated KPCY allograft mice. Scale bars, 100 μm. Quantification is presented in Extended Data Fig. 2u. Source Data

**Extended Data Fig. 2:**
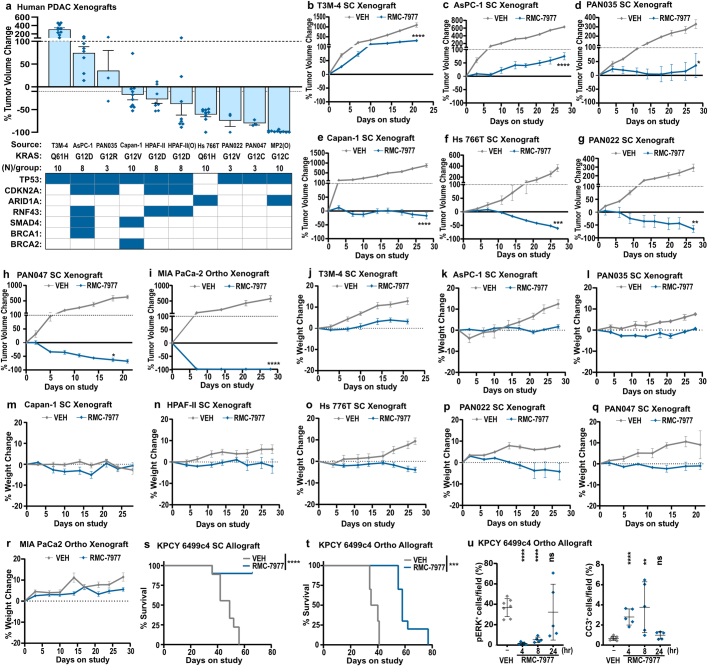
**(a-r)** Human PDAC xenograft models from Fig. 2a. **(a)** Waterfall plot showing tumor volume change from baseline in RMC-7977 treated tumors. Error bars indicate ±s.e.m. Table shows selected genotypes for the xenograft panel with the row above indicating the *KRAS* mutation and number of animals. Present co-mutations in each model shown as blue squares in the table. **(b-i)** Tumor growth curves for indicated xenograft models from (a), shown as percent tumor volume change from baseline over time. Vehicle and RMC-7977 groups were compared by 2-way repeated measures ANOVA on the last measurement day of the vehicle group (*, p < 0.05; **, p < 0.01; ***, p < 0.001; ****, p < 0.0001). Error bars indicate ±s.e.m. **(j-r)** Tolerability of RMC-7977 as assessed by percent animal body weight change from baseline over time, for indicated xenograft models from (a). Error bars indicate ±s.e.m. **(s,t)** Kaplan-Meier survival analysis comparing RMC-7977 and Vehicle treatment arms in **(s)** subcutaneous and **(t)** orthotopic KPCY 6499c4 allograft models (***, p < 0.001; ****, p < 0.0001). **(u)** Quantification of phospho-ERK^T202/Y204^ (left) and CC3 (right) staining in tumors from orthotopic KPCY 6499c4 allograft model collected at indicated timepoints post single dose of Vehicle or RMC-7977. Analysis of IHC images based on 10 fields of view and plotted as average per tissue section. Results were compared by two-tailed Student’s unpaired t-test (**, p < 0.01; ****, p < 0.0001). Error bars indicate ±s.d. Source Data

We next assessed the anti-tumour activity of RMC-7977 in vivo, beginning with a panel of seven human PDAC cell line-derived xenograft (CDX) and three patient-derived xenograft (PDX) models, implanted either subcutaneously or orthotopically in the pancreas of immune-deficient mice (see Supplementary Table 1 for information on tumour models). RMC-7977 was administered orally at a daily dose of 10 mg kg^−1^ for 21–28 days and resulted in significant anti-tumour activity in all 10 models. Tumour regressions were observed in 7 out of 10 models and ranged from 30% to 98% relative to baseline volume (Fig. 2a–d and Extended Data Fig. 2a–i). Of note, the *KRAS*^Q61H^ mutant Hs 766T line, which showed lower sensitivity in vitro (Fig. 1b), was among the most responsive in the CDX setting (Fig. 2a). Notably, RMC-7977 was well-tolerated in all ten models, with the treated mice generally exhibiting stable body weight over time (Fig. 2e and Extended Data Fig. 2j–r).

To assess RMC-7977 activity in models with an intact immune system, we generated cell line-derived allograft (CDA) models from a *Kras*^*LSL.G12D/+*^;*Trp53*^*R172H/+*^;*Pdx1-cre*^*tg/+*^;*Rosa26*^*LSL.YFP/+*^ (KPCY) mouse PDAC cell line implanted either subcutaneously or orthotopically in immunocompetent C57Bl/6 mice. In this setting, RMC-7977 still diminished tumour growth and extended overall survival, although without inducing significant regressions (Fig. 2f and Extended Data Fig. 2s,t). Pharmacodynamic analyses of the orthotopic CDA model showed reduced pERK levels and increased apoptosis in the tumours 4 h after treatment, followed by recovery of pathway activity at 24 h (Fig. 2g and Extended Data Fig. 2u). Quantitative analysis of RMC-7977 levels in matched tumour tissue samples found that the restoration of RAS–MAPK activity in these tissues was consistent with the observed tumour half-life of 3.5 h in this model (Supplementary Table 2). Together, these results demonstrate anti-tumour activity by RMC-7977 across a range of implanted PDAC models.

## The pharmacology of RAS dependence

**Fig. 3:**
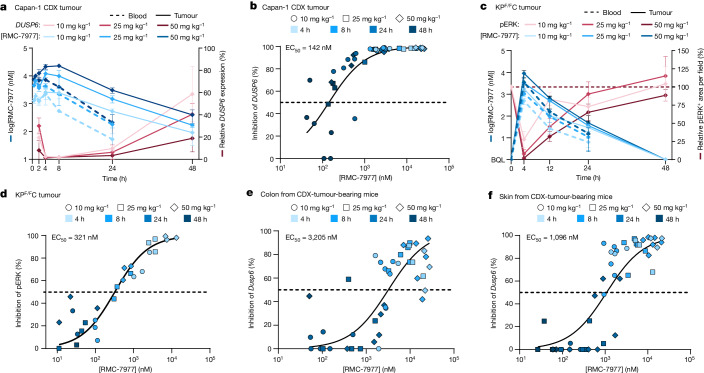
Pharmacology of RAS dependence. **a**,**b**, Capan-1 subcutaneous xenograft mice were treated with a single dose of vehicle or RMC-7977 at 10 mg kg^−1^, 25 mg kg^−1^ or 50 mg kg^−1^. Tissues were collected at indicated timepoints (*n* = 3–6 per timepoint and dose). **a**, Pharmacokinetic–pharmacodynamic study in the Capan-1 xenograft model. Pharmacokinetic profile is shown as RMC-7977 concentration in tumours (solid blue lines) and blood (dashed blue lines) over time. Pharmacodynamic response is shown as relative change in *DUSP6* mRNA expression (solid red lines) over time. Shades of blue or red represent the three tested doses of RMC-7977. Data are mean ± s.d. **b**, Pharmacokinetic–pharmacodynamic relationship between RMC-7977 concentration and inhibition of *DUSP6* expression in tumours. **c**,**d**, Tumour-bearing KP^F/F^C mice were treated with a single dose of vehicle or RMC-7977 at 10 mg kg^−1^, 25 mg kg^−1^ or 50 mg kg^−1^. Tissues were collected at indicated timepoints (*n* = 3 per timepoint and dose). **c**, Pharmacokinetic–pharmacodynamic relationship in the KP^F/F^C mouse model. Pharmacokinetic response is shown as RMC-7977 concentration in tumours (solid blue lines) and blood (dashed blue lines) over time. Pharmacodynamic response is shown as relative change in pERK^T202/Y204^-positive IHC staining in tumours (solid red lines) over time. Shades of blue or red represent different doses. Data are mean ± s.d. BQL, below quantifiable limit. **d**, Pharmacokinetic–pharmacodynamic relationship between RMC-7977 concentration and pERK^T202/Y204^ inhibition in tumours. **e**,**f**, CDX tumour-bearing mice were treated with a single dose of vehicle or RMC-7977 at 10 mg kg^−1^, 25 mg kg^−1^ or 50 mg kg^−1^. Tissues were collected at indicated timepoints (*n* = 3–6 per timepoint and dose). Pharmacokinetic–pharmacodynamic relationship between RMC-7977 concentration and inhibition of *Dusp6* expression in normal colon (**e**) and skin (**f**). **b**,**d**–**f**, A three-parameter sigmoidal exposure response model was fitted to the data to derive EC_50_ values. Source Data

We next carried out a detailed and quantitative analysis of the pharmacological profile of RMC-7977 in the PDAC setting. We first examined the association of drug concentration and RAS pathway inhibition in a representative human CDX model of PDAC (Capan-1 (*KRAS*^G12V^)). We measured the response of tumour cells to treatment with a single dose of 10, 25 or 50 mg kg^−1^ RMC-7977 using quantitative PCR with reverse transcription (RT–qPCR) for human *DUSP6*, a RAS–MAPK pathway transcriptional target. *DUSP6* levels were effectively inhibited for 24–48 h after the treatment and pathway inhibition was tightly associated with concentrations of RMC-7977 in tumours (Fig. 3a,b; half-maximal effective concentration (EC_50_) = 142 nM in Capan-1 CDX tumours), indicating that the local drug concentration is a critical determinant of biochemical activity. We also observed somewhat prolonged RMC-7977 exposure in Capan-1 xenograft tumours, with an approximately threefold increase in overall exposure (AUC_0–48_) compared with that in blood (Supplementary Table 2).

**Extended Data Fig. 3:**
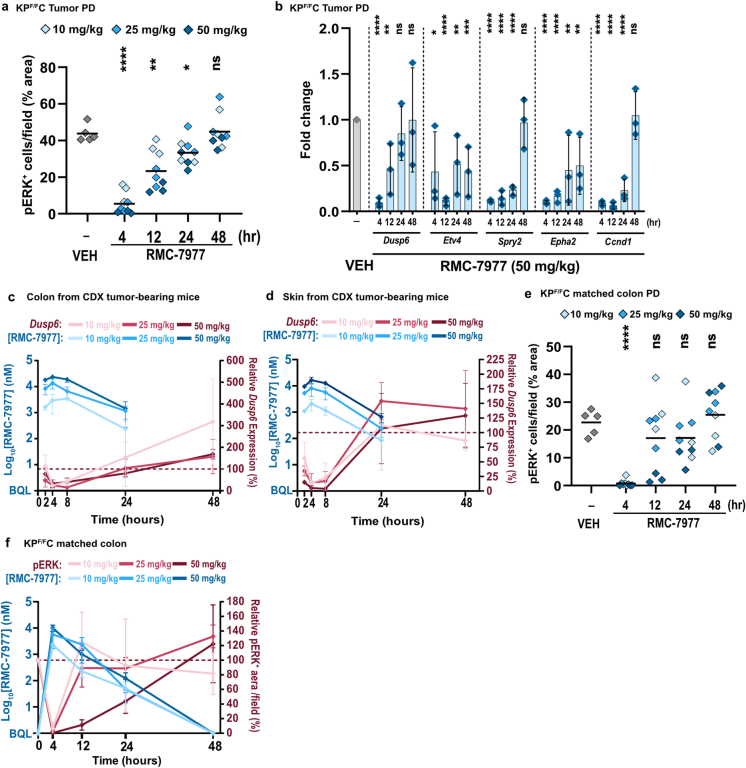
Analysis was ran on tissues collected from mice in Fig. 3. **(a)** Quantification of phospho-ERK^T202/Y204^ IHC staining of tumors isolated from Vehicle and RMC-7977-treated KP^F/F^C mice, at indicated timepoints post single dose. **(b)** qRT-PCR analysis was ran on tumors collected from KP^F/F^C mice treated with a single dose of Vehicle or RMC-7977 (50mg/kg). Plot shows relative expression of five MAPK pathway signature genes over time. Results were compared by two-tailed Student’s unpaired t-test (*, p < 0.05; **, p < 0.01; p < 0.001; ****, p < 0.0001). Error bars indicate ±s.d. **(c,d)** PK/PD in the colons and skin isolated from CDX tumor-bearing mice. Pharmacokinetic response shown as RMC-7977 concentration in **(c)** colon and **(d)** skin (solid blue lines) over time. Pharmacodynamic response shown as relative change in *Dusp6* expression in colon or skin (red solid lines) over time. Shades of blue or red represent three tested doses. Error bars indicate ±s.d. **(e)** Quantification of phospho-ERK^T202/Y204^ IHC staining of colons isolated from Vehicle and RMC-7977-treated KP^F/F^C mice, at indicated timepoints post single dose. **(f)** PK/PD of RMC-7977 in the colons isolated from KP^F/F^C mice. Pharmacokinetic response shown as RMC-7977 concentration in colon (solid blue lines) over time. Pharmacodynamic response shown as relative change in phospho-ERK^T202/Y204^ positive IHC staining (red solid lines) over time. Shades of blue or red represent three tested doses. Error bars indicate ±s.d. **(a,e)** Analysis of IHC images based on 10 fields of view and plotted as average per tissue section. Shades of blue represent three tested doses. Results were compared by two-tailed Student’s unpaired t-test (*, p < 0.05; **, p < 0.01; ****, p < 0.0001). Source Data

The delivery of drugs to autochthonous PDAC tumours can be impeded by their expansive desmoplastic stroma, which both suppresses tumour vascularity and impedes diffusion^26,27^. To assess whether the pharmacology of RMC-7977 permits effective targeting of RAS-GTP in the context of a native tumour microenvironment, we utilized *Kras*^*LSL.G12D/+*^;*Trp53*^*flox/flox*^;*Pdx1-cre*^*tg/+*^ (KP^F/F^C) mice^28^, a genetically engineered model that rapidly develops autochthonous *Kras*^G12D^ mutant PDAC. We observed that the KP^F/F^C model was somewhat less sensitive to pathway modulation by RMC-7977 and exhibited earlier pathway recovery compared with the Capan-1 CDX model. Consequently, KP^F/F^C mice required higher doses (25 to 50 mg kg^−1^) to achieve maximal and durable suppression of RAS–MAPK signalling, as measured by decreases in tumour pERK expression (Fig. 3a,c and Extended Data Fig. 3a). We also noted that the pharmacokinetics of pERK inhibition closely paralleled the decrease in RNA expression of several MAPK pathway downstream target genes (Extended Data Fig. 3b). Examining the pharmacokinetic profile of RMC-7977 in KP^F/F^C mice, we found that exposure in the blood and PDAC tumours was lower than in CDX tumour-bearing BALB/c immune-deficient mice, comparable to that in the KPCY CDA model. This implies that strain- and model-specific variables could both contribute to the exposure and relative activity of RMC-7977 (Fig. 3a,c and Supplementary Table 2). We also observed a shorter half-life (*t*_1/2_) of RMC-7977 in KP^F/F^C tumours compared with CDX tumours and concordantly faster recovery of pERK levels (Fig. 3c,d). Notably, the tight relationship between RMC-7977 concentration and pathway suppression observed in CDX models was maintained in this autochthonous model (Fig. 3d, EC_50_ = 321 nM). Moreover, in all model systems tested, concentrations of RMC-7977 were higher in tumour tissues than in blood (*K*_p_ = AUC_tumour_/AUC_blood_ > 1; Supplementary Table 2), indicating that the pharmacology of RMC-7977 can overcome the biophysical constraints to drug delivery imposed by the desmoplastic tumour microenvironment. On the basis of this result and the reproducible tumour drug concentration–response (pharmacokinetic–pharmacodynamic) relationship across models, we predicted that daily or alternate-day dosing schedules would yield an effective and metronomic pattern of RAS–MAPK pathway suppression in pancreatic tumours.

To assess the effect of RMC-7977 on normal tissues, we measured its effect on mouse *Dusp6* mRNA levels (using RT–qPCR) in normal colon and skin, two proliferative tissues that are known to rely on RAS signalling for self-renewal^29,30^. The EC_50_ of RMC-7977 was 3,205 nM in colon and 1,096 nM in the skin of CDX tumour-bearing mice, representing reductions in potency of around 22-fold and 8-fold, respectively, compared with Capan-1 xenograft tumours (EC_50_ = 142 nM; Fig. 3b,e,f and Extended Data Fig. 3c,d). We also found that pERK suppression via RMC-7977 in colon tissues from the KP^F/F^C cohort was rapidly restored to baseline at the 10 and 25 mg kg^−1^ dose levels, whereas pathway suppression was more prolonged in tumours at these doses (Fig. 3c and Extended Data Fig. 3e, f). Together these data highlight a marked difference in the potency and kinetics of RMC-7977-mediated pathway modulation in normal tissues expressing wild-type RAS compared with *KRAS*^G12X^-driven PDAC tumours^29^.

**Fig. 4:**
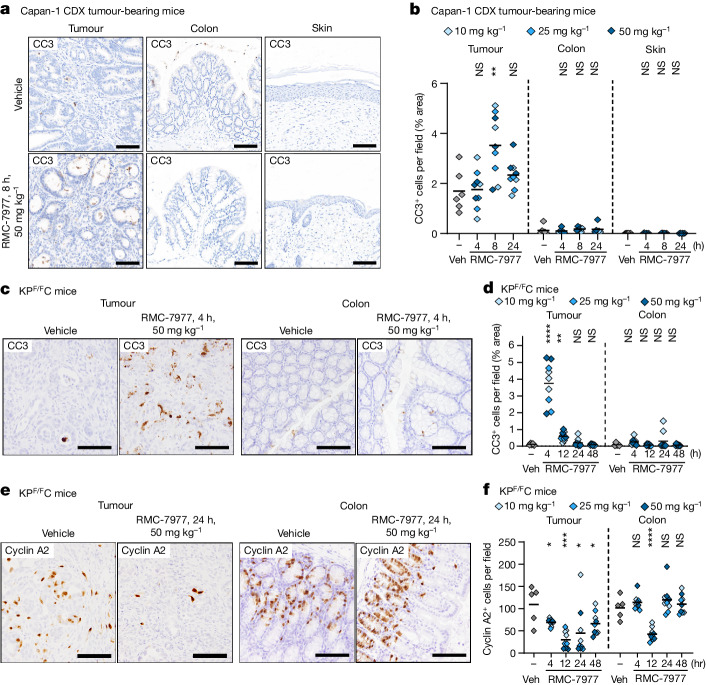
Inhibition of RAS induces pancreatic tumour-selective apoptosis. **a**,**b**, Capan-1 subcutaneous xenograft mice (from Fig. 3) were treated with a single dose of vehicle (veh) or RMC-7977 at 10 mg kg^−1^, 25 mg kg^−1^ or 50 mg kg^−1^. Tissues were collected at indicated timepoints (*n* = 3–6 per timepoint and dose). **a**, Representative IHC of tumours, colon and skin from the Capan-1 xenograft model collected at 8 h after a single dose of vehicle or RMC-7977, stained for CC3. Scale bars, 100 μm. **b**, Quantification of CC3 staining in tumours, colon, and skin. **b**–**f**, Tumour-bearing KP^F/F^C mice were treated with a single dose of vehicle or RMC-7977 at 10 mg kg^−1^, 25 mg kg^−1^ or 50 mg kg^−1^. Tissues were collected at indicated timepoints (*n* = 3 per timepoint and dose). **c**, Representative IHC of KP^F/F^C tumours and colons collected at 4 h after a single dose of vehicle or RMC-7977, stained for CC3. Scale bars, 50 μm. **d**, Quantification of CC3 staining in tumours and colons. **e**, Representative IHC of KP^F/F^C tumours and colons collected at 24 h after a single dose of vehicle or RMC-7977, stained for cyclin A2. Scale bars, 50 μm. **f**, Quantification of cyclin A2 staining in tumours and colons. **b**,**d**,**f**, Analysis of IHC based on ten fields of view and plotted as the average per tissue section. Shades of blue represent the tested doses. Results were compared by two-tailed unpaired Student’s *t*-test. Source Data

Next, we assessed how normal and tumour tissues responded to RAS-GTP inhibition by quantifying markers of cell proliferation and apoptosis. In Capan-1 CDX tumours, we observed a notable increase in CC3^+^ apoptotic cells, peaking at 8 h post-dose, relative to vehicle controls. By contrast, few apoptotic cells were observed in the colon or the skin from tumour-bearing mice at any timepoint (Fig. 4a,b). In KP^F/F^C tumours, we observed a sharp, approximately fourfold spike in CC3^+^ apoptotic cells at 4 h following a single dose of RMC-7977, which was not observed in the matched colon tissues from these mice (Fig. 4c,d). In both the KP^F/F^C and CDX models, the kinetics of apoptosis initiation in tumours aligned with the onset of full pERK inhibition. By contrast, CC3^+^ apoptosis in normal tissues was negligible or absent across all doses and timepoints, even at times when pERK was fully inhibited.

Finally, we detected a sustained decrease in cell proliferation in KP^F/F^C tumours (as measured by IHC for cyclin A2) beginning 4 h after treatment with RMC-7977, with maximal inhibition maintained at 12 and 24 h and a partial recovery at 48 h (Fig. 4e,f). By contrast, in matched colon tissue from these KP^F/F^C mice, we observed only a transient decrease in proliferation at 12 h, with cyclin A2 levels fully restored by 24 h (Fig. 4e,f). Thus, the overall proliferation of this self-renewing normal tissue was minimally affected compared with the sustained anti-proliferative response observed in PDAC tissues. Together, these results demonstrate key differences in how RAS wild-type normal tissues react and adapt to metronomic RAS inhibition with a broad-spectrum RAS inhibitor compared with tumours driven by mutant KRAS, providing a rational basis for the tumour selectivity of RAS inhibition in PDAC.

## Efficacy and tolerability of RMC-7977

**Fig. 5:**
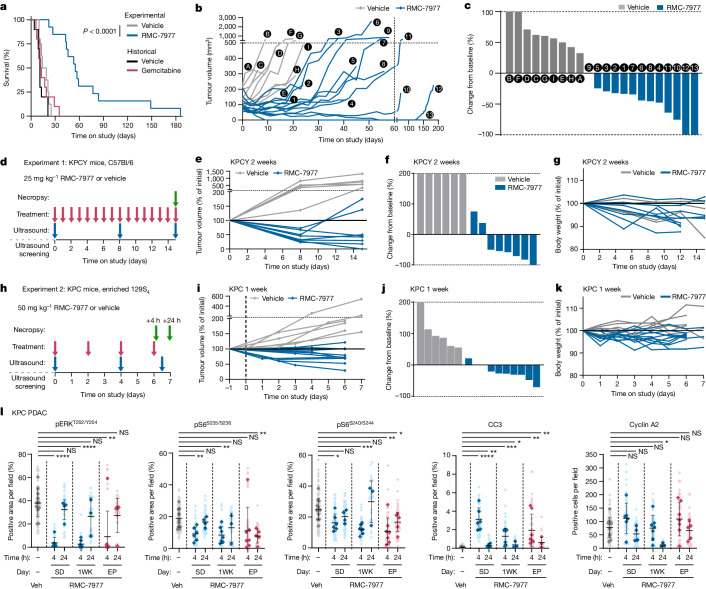
RMC-7977 inhibits tumour growth and extends survival in autochthonous models of PDAC. **a**–**c**, KPC mice treated with vehicle (*n* = 9) or RMC-7977 (50 mg kg^−1^ orally, on alternating days; *n* = 13) until endpoint criteria were met. **a**, Kaplan–Meier survival analysis comparing RMC-7977 to vehicle, and historical data from gemcitabine and vehicle treatment arms. **b**, Tumour growth rates calculated from longitudinal tumour volumes. **c**, Waterfall plot showing best response for each tumour relative to initial volume. **b**,**c**, Letters represent individual vehicle-treated mice and numbers represent RMC-7977 treated mice. **d**, Treatment scheme for KPCY mice (C57Bl/6 background) with vehicle (*n* = 6) or RMC-7977 (25 mg kg^−1^ orally, once daily; *n* = 8) for 15 days. **e**, Tumour growth curves for mice in the experiment depicted in **d**. **f**, Waterfall plot showing percentage change in tumour volume compared with baseline after 15 days of treatment. **g**, RMC-7977 tolerability as assessed by body weight change from baseline over time. **h**, Treatment scheme for KPC mice (129S4/SvJae background) with vehicle (*n* = 6) or RMC-7977 (50 mg kg^−1^, orally, on alternating days; *n* = 11) for 1 week. Tissues collected at 4 h (*n* = 7) or 24 h (*n* = 4) after the last dose. **i**, Tumour growth curves for mice in the experiment depicted in **h**. **j**, Waterfall plot showing percent change in tumour volume compared with baseline after one week of treatment. **k**, RMC-7977 tolerability assessed by body weight change from baseline over time. **l**, IHC analysis of KPC tumours treated with vehicle or RMC-7977 for the indicated time, with tissues collected either at 4 h or 24 h after the last dose. Tumours were stained for pERK^T202/Y204^, pS6^S235/236^, pS6^S240/244^, CC3 and cyclin A2. Quantification of IHC was based on ten fields of view (light shade), averaged per tumour (dark shade) and means were compared by two-tailed unpaired Student’s *t*-test. Error bars indicate ±s.d. Source Data

**Extended Data Fig. 4:**
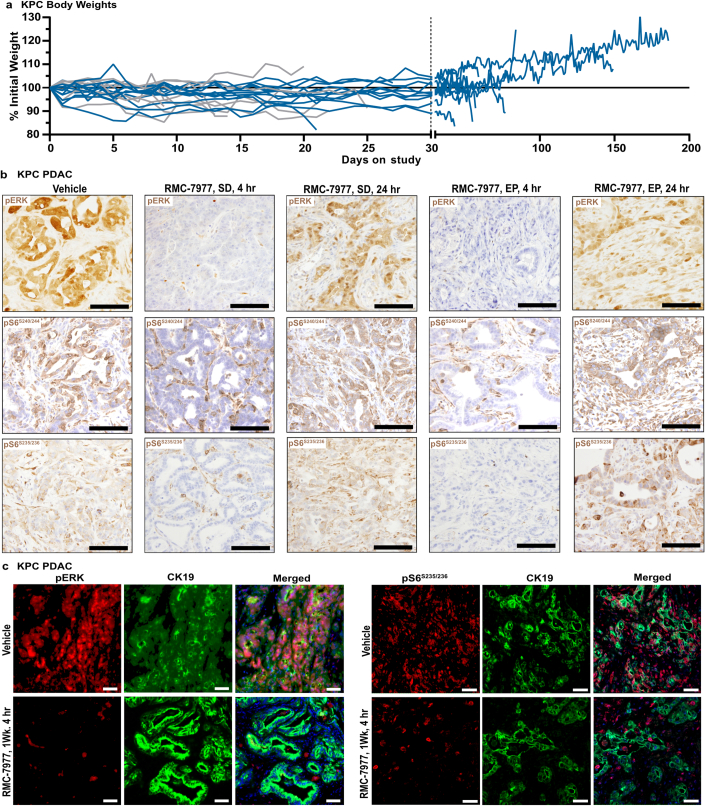
**(a)** KPC mice (from Fig. 5) treated with Vehicle (n = 9) or RMC-7977 (50 mg/kg, p.o., q.o.d., n = 13) until endpoint criteria were met. Tolerability of RMC-7977 in KPC mice as assessed by percent animal body weight change from baseline over time. **(b,c)** KPC mice (from Fig. 5) were treated with Vehicle (n = 10) or RMC-7977 (n = 31) for indicated time, with tissues collected either at 4 or 24 h post last dose. **(b)** Representative IHC images of KPC tumors collected at indicated timepoints, stained for phospho-ERK^T202/Y204^, phospho-S6^S235/236^ and phospho-S6^S240/244^. Scale bars = 100 μm. **(c)** Representative IF images of KPC tumors collected at indicated timepoints, stained for phospho-ERK^T202/204^ (left panel, red), phospho-S6^S235/236^ (right panel, red) and CK19 (green), and counterstained with DAPI (blue). Scale bars = 50 μm. Source Data

To evaluate the anti-tumour activity of RMC-7977 in clinically predictive models of human PDAC, we first performed an interventional survival study in tumour-bearing *Kras*^*LSL.G12D/+*^*;Trp53*^*LSL.R172H/+*^*;Pdx1-cre*^*tg/+*^ (KPC) mice. Kaplan–Meier analysis of overall survival showed a 3.5-fold increase in median survival in the RMC-7977-treated cohort compared with controls (Fig. 5a), exceeding the most effective therapy reported in the KPC model^31,32^. By comparison, cytotoxic chemotherapies such as gemcitabine—and monotherapies in general—do not significantly alter survival in this model^26^. Longitudinal, high-resolution 3D ultrasound imaging^33^ revealed that mice treated with 50 mg kg^−1^ RMC-7977 (administered on alternate days) exhibited tumour stabilizations or regressions. This included two mice that lived several months on treatment after their tumours became undetectable by ultrasound. By contrast, tumours in vehicle-treated mice uniformly exhibited progressive growth (Fig. 5b,c). The body weights of KPC mice treated with RMC-7977 were similar to those of control KPC mice (Extended Data Fig. 4a).

Next, we carried out short-term intervention studies at multiple timepoints in two variants of the KPC model. Performed independently at separate institutions, the first study treated a YFP lineage-traced version of the KPC model (KPCY), on a pure C57Bl/6 background, with daily 25 mg kg^−1^ RMC-7977, for 2 weeks (Fig. 5d–g). The second study treated KPC mice, on a background enriched for 129S_4_/SvJae, with 50 mg kg^−1^ RMC-7977 (administered on alternate days), for one week (Fig. 5h–k). In both experiments, RMC-7977 treatment induced regressions in most tumours, with little effect on body weight (Fig. 5e–g, i–k).

To characterize the pharmacodynamic responses of KPC tumours and normal tissues to RMC-7977-mediated inhibition of active RAS, we performed IHC and RT–qPCR analyses on samples from additional KPC mice treated with a single dose of RMC-7977 or vehicle (SD), from treated mice in the one-week KPC study (1WK), and on endpoint tumour samples collected from mice that relapsed on treatment (resistant) in the KPC survival study (EP)(summarized in Supplementary Table 3). Within each group of mice, mice were euthanized either 4 h or 24 h after dosing to capture the dynamic changes associated with metronomic pathway inhibition.

**Extended Data Fig. 5:**
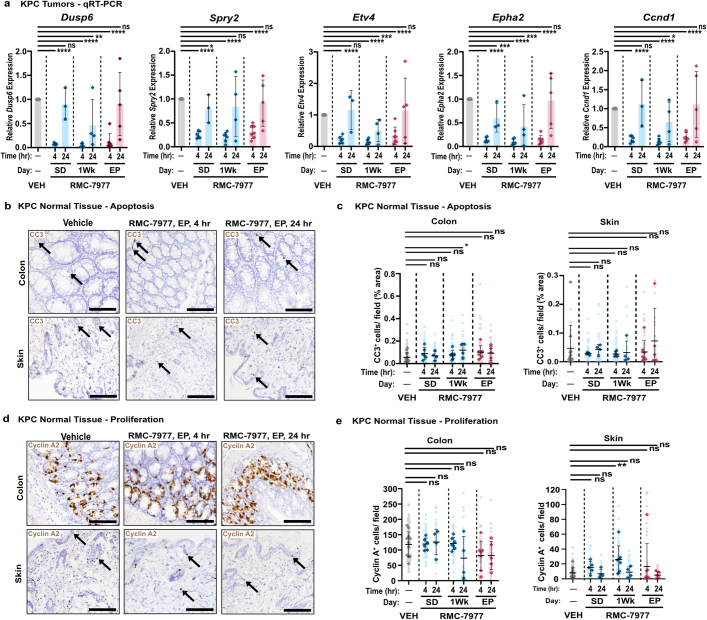
**(a-e)** KPC mice (from Fig. 5) were treated with Vehicle (n = 11) or RMC-7977 (50 mg/kg, n = 33) for indicated time, with tissues collected either at 4 or 24 h post last dose **(a)** qRT-PCR analysis was run on RNA isolated from KPC tumors. Plots show relative expression of five MAPK pathway signature genes at indicated timepoints. Results were compared by two-tailed Student’s unpaired t-test (*, p < 0.05; **, p < 0.01; p < 0.001; ****, p < 0.0001). Error bars indicate ±s.d. **(b)** Representative IHC images of KPC colon and skin collected at endpoint and stained for CC3. Scale bars = 100 μm. **(c)** Quantification of CC3 IHC staining in colon, and skin. **(d)** Representative IHC images of KPC colon and skin collected at endpoint and stained for Cyclin A2. Scale bars = 100 μm. **(e)** Quantification of Cyclin A2 IHC staining in colon and skin. **(c,e)** Quantification of IHC images was based on 10 fields of view (light shade), averaged per tissue section (dark shade) and means were compared by two-tailed Student’s unpaired t-test (*, p < 0.05; **, p < 0.01). Error bars indicate ±s.d. Source Data

Quantification of pERK IHC staining on tumour samples from SD mice showed that the effects of a single dose of RMC-7977 in KPC tumours were consistent with our earlier observations in the KP^F/F^C model, reflecting near-complete inhibition of RAS–MAPK signalling 4 h after treatment followed by restoration by 24 h (Fig. 5l). A similar pattern was also observed for S6RP phosphorylation at both the S235/S236 and S240/S244 sites; however, for these two epitopes, inhibition was largely restricted to the malignant epithelial compartment, indicating that pS6 is regulated by alternative pathways in stromal cells (Extended Data Fig. 4b,c). Crucially, the metronomic pattern of pathway inhibition was also apparent in the 1WK and EP samples, demonstrating a persistent and cyclical pharmacodynamic response to RMC-7977 treatment over time. These data were supported by parallel analyses of the same tumours using RT–qPCR, showing that five MAPK pathway target genes were similarly regulated at the transcriptional level (Extended Data Fig. 5a).

Next, we examined the cellular responses of KPC tumours at each timepoint. The wave of apoptosis that was observed in KP^F/F^C mice 4 h after a single dose of RMC-7977 (Fig. 4d) was also observed in SD, 1WK and EP KPC mouse samples collected 4 h after treatment (Fig. 5l), suggesting that each successive dose of RMC-7977 induces an additional wave of apoptosis. KPC tumours also showed a trend towards reduced proliferation following a single dose of RMC-7977, which was pronounced and significant after a week of treatment. However, in contrast to the persistent induction of apoptosis, proliferation was no longer deeply suppressed at 24 h post-dosing in response to RAS inhibition in EP tumours (Fig. 5l, cyclin A2; see ‘Mechanisms of resistance to RMC-7977’).

Finally, to assess the effects of long-term treatment on normal tissues, we performed IHC on colon and skin samples from the SD, 1WK and EP KPC mice. Effects of RMC-7977 on apoptosis (CC3) and proliferation (cyclin A2) were absent or negligible at all timepoints in both tissues (Extended Data Fig. 5b–e). More broadly, in a blinded histopathological review of haematoxylin and eosin-stained liver, intestines, lungs, kidneys and skin in all KPC mice from the study, the only treatment-associated histopathological feature that we detected was a modest increase in apoptosis in the proximal intestines of half of the treated mice, although this was not associated with diarrhoea or weight loss. Together, these preclinical findings have important and positive implications for the translation of multi-selective RAS-GTP inhibition in patients with PDAC and potentially other types of RAS-addicted cancers.

## Mechanisms of resistance to RMC-7977

Emerging preclinical and clinical data demonstrate a diverse range of potential mechanisms through which tumours may acquire resistance to mutation-selective RAS inhibitors. In the majority of cases, tumours overcome mutation-selective RAS inhibition through reactivation of RAS–MAPK signalling, either through the emergence or outgrowth of clones with second-site RAS mutations, through amplification of pathway members, or through compensatory signalling mechanisms^9,10,34,35^. To assess the potential mechanisms of resistance to a multi-selective RAS-GTP inhibitor, we analysed the EP KPC pancreatic tumours that relapsed following initial responses in the survival study, developing resistance while on continuous RMC-7977 treatment. Of 7 evaluable EP KPC tumours (for example, those that were collected 4 h after the final dose, when RAS signalling is suppressed in naive tumours), 6 tumours (86%) continued to show full inhibition of pERK expression (Fig. 5l), thereby excluding several classes of mechanisms that reactivate RAS–MAPK signalling. The same six tumours also exhibited continued inhibition of pS6^S235/S236^ and pS6^S240/S244^, excluding mechanisms that primarily affect the PI3K–mTOR arm of RAS signalling (Fig. 5l). Of interest, the mouse hosting the one EP tumour that was refractory to pERK modulation owing to apparently reactivated RAS pathway signalling (tumour 12 in Fig. 5b) survived for more than half a year on treatment, longer than any other mouse in the study.

**Fig. 6:**
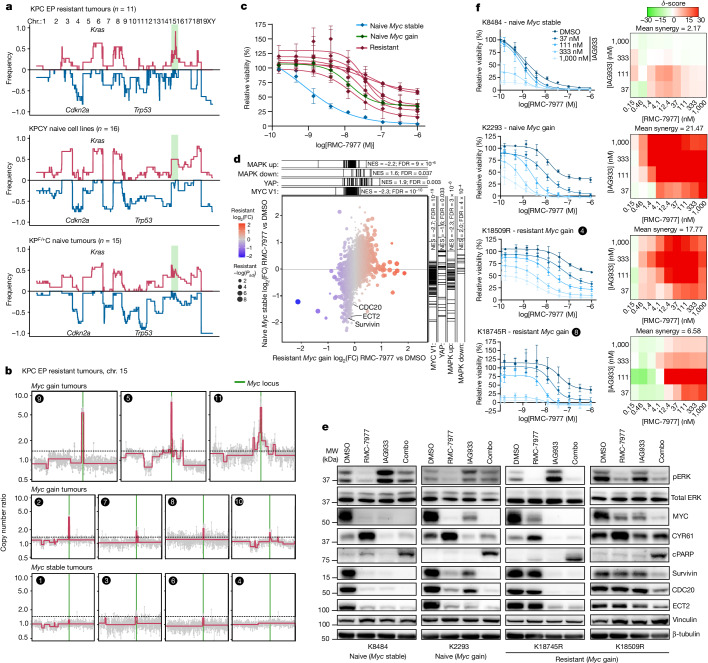
Resistance to RMC-7977 predominantly arises independently of MAPK activity. **a**, CNV analysis of DNA isolated from epithelial cells from RMC-7977-resistant KPC tumours (top), KPCY tumour-derived cell lines (middle) and KP^F/+^C naive tumours (bottom). The region highlighted in green marks chromosome 15, which includes the *Myc* locus. Chr., chromosome. **b**, CNV plots showing chromosome 15 (chr. 15) in RMC-7977-resistant KPC tumours. The vertical green line marks the *Myc* locus. The horizontal dashed line indicates the threshold to be called as a gain. Numbers indicate tumour identity from Fig. 5. **c**–**f**, Cell lines derived from RMC-7977-resistant or naive KPC tumours. **c**, Cell lines treated with DMSO or indicated concentrations of RMC-7977 for 3–5 days. Data are mean ± s.d. of three biological replicates normalized to DMSO control. Values reproduced from DMSO control (RMC-7977 alone) from Fig. 6f and Extended Fig. 7a. **d**, Mass spectrometry-based proteomic analysis comparing the effects of RMC-7977 and DMSO treatment in resistant K18509R (*Myc* gain) and naive K8484 (*Myc* stable). Differential protein expression signatures within each line were analysed for enrichment of published functional gene sets (MAPK, MYC and YAP–TAZ). FC, fold change; FDR, false discovery rate; NES, normalized enrichment score. **e**, Western blot analyses of two RMC-7977-resistant (K18745R and K18509R) and two naive (K8484 and K2293) cell lines treated with DMSO, RMC-7977 (100 nM), IAG933 (1 μM) or the combined treatment (combo) for 24 h. Vinculin and β-tubulin were used as loading controls. **f**, Cell lines described in **e** were treated with indicated concentrations of DMSO, RMC-7977, IAG933 or the combined treatment. Right, dose–response matrices show combination synergy based on cell viability at different dose pairs. Left, viability of cell lines treated with a range of RMC-7977 concentrations in combination with the indicated concentration of IAG933. Data are mean ± s.d. of 3–4 biological replicates normalized to DMSO control. Source Data

**Extended Data Fig. 6:**
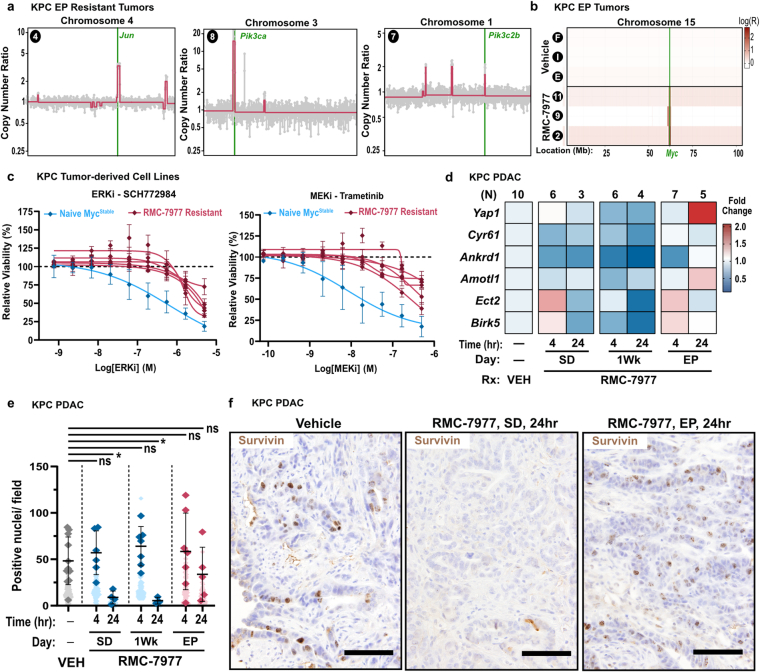
**(a)** CNV analysis of DNA isolated from epithelial cells of RMC-7977 resistant KPC tumors. Green lines mark amplified regions of interest. Numbers represent matched tumors from Fig. 5. **(b)** Targeted sequencing of *Myc* locus in RMC-7977 resistant KPC tumors and KPC Vehicle tumors. Letters and numbers represent matched tumors from Fig. 5. **(c)** Cell lines derived from RMC-7977 resistant or naive KPC tumors, treated with DMSO or indicated concentrations of SCH772984 (ERKi, left) or Trametinib (MEKi, right) for 3 days. Data points represent the mean of 3 replicates normalized to DMSO control. Error bars indicate ± s.d. RMC-7977 resistance and *Myc* status are indicated by curve colors. **(d-f)** KPC mice (from Fig. 5) were treated with Vehicle or RMC-7977 (50 mg/kg) for indicated time, with tissues collected either at 4 or 24 h post last dose. **(d)** qRT-PCR analysis was run on RNA isolated from KPC tumors. Heatmap shows changes in expression of YAP/TAZ/TEAD pathway signature genes in RMC-7977 mice relative to Vehicle controls. **(e)** Quantification of Survivin IHC staining of KPC tumors. **(f)** Representative IHC images of KPC Vehicle tumors and RMC-7977 tumors collected 24 h post single dose or at endpoint. Scale bars = 100 μm. Source Data

To examine a broader range of potential resistance mechanisms, we used laser capture microdissection to extract DNA from RMC-7977 treated EP tumours (all tumours available at the time of evaluation, tumours 1–11 in Fig. 5b) and performed sparse genome copy number variation (CNV) analysis, comparing both to a set of 5 vehicle-treated EP tumours as well as to historical reference samples comprising 15 KP^F/+^C pancreatic tumours^36^ (KPC with a heterozygous conditional null allele) and cell lines from 16 KPCY pancreatic tumours^37^. Overall, the global genomic profiles of RMC-7977-resistant KPC tumours closely reflected those of the reference samples (Fig. 6a). However, 1 prominent exception was apparent: 7 out of the 11 (64%) RMC-7977-resistant tumours exhibited focal copy number gains in *Myc*, an oncogenic transcription factor that receives mitogenic signals from the RAS pathway (Fig. 6b). By comparison, only 3 out of 36 (8%) control KPC or KPCY samples exhibited gains at the *Myc* locus (*P* = 0.0003, Fisher’s exact test). An additional RMC-7977-resistant tumour exhibited a focal gain in *Jun*, a canonical member of the AP1 transcription factor complex that acts downstream of the RAS–MAPK pathway to drive proliferation (Extended Data Fig. 6a)—*Jun* copy number gains have rarely been reported in PDAC or other carcinomas. Finally, two RMC-7977-resistant KPC tumours exhibited focal gains of genes encoding PI3K family members (*Pik3ca* in tumour 8 and *Pik3c2b* in tumour 7), in both cases co-occurring with gains in *Myc* (Extended Data Fig. 6a,b). Targeted resequencing of the *Myc* locus in a subset of EP KPC tumours (three treated with vehicle and three treated with RMC-7977) provided orthogonal validation of the presence of *Myc* copy number gains in RMC-7977-treated EP tumours (Extended Data Fig. 6b). Together, these data suggest that broad RAS-GTP inhibition with a tri-complex inhibitor such as RMC-7977 forces KPC pancreatic tumours down a narrower evolutionary path to resistance compared with mutation-specific RAS inhibitors (with which the RAS pathway is frequently reactivated), with the most prominent mechanisms affecting transcription factors downstream of RAS–MAPK signalling.

To further investigate the relevance of *Myc* copy number gains, we established cell lines from six RMC-7977-resistant tumours, of which four were confirmed to harbour *Myc* gains (see Supplementary Table 3). All six lines from resistant tumours were less sensitive to RMC-7977 than a control KPC cell line (K8484) derived from a treatment-naive KPC tumour and confirmed not to have a gain at the *Myc* locus (*Myc* stable) (Fig. 6c). Another treatment-naive KPC cell line (K2293) was identified with a spontaneous *Myc* gain and found to have correspondingly lower sensitivity to RMC-7977. Notably, all lines from RMC-7977-resistant tumours also exhibited resistance to inhibitors of the downstream MAPK pathway effectors MEK and ERK (Extended Data Fig. 6c). We then used mass spectrometry-based proteomics to compare the effects of RMC-7977 versus DMSO treatment in one *Myc*-gain resistant line (K18509R) and the naive *Myc*-stable line (K8484) (Fig. 6d) and queried the differential protein expression signatures within each line for enrichment of functional gene sets. Although both lines showed downregulation of an experimental MAPK pathway gene expression signature (Methods) upon RMC-7977 treatment, many more proteins were differentially expressed in response to RMC-7977 in the *Myc*-stable than in the *Myc*-gain resistant lines (Fig. 6d, compare variance along vertical and horizontal axes).

Among the gene sets that diverged between the two cell lines, a YAP–TAZ response signature stood out as being reduced by RMC-7977 in the sensitive line but activated in the resistant line (Fig. 6d). This led us to hypothesize that the YAP–TAZ pathway has a role in supporting MYC-driven resistance to RMC-7977. To test whether the YAP–TAZ pathway was modulated in response to RMC-7977 treatment in vivo, we first performed RT–qPCR analyses on tumour samples from the SD, 1WK and EP KPC mice. We found that expression of *Yap1* and several established YAP target genes (*Cyr61*, *Ankrd1*, *Amotl2*, *Birc5* and *Ect2*) were initially downregulated in response to RMC-7977 treatment, particularly after 1 week of treatment (Extended Data Fig. 6d, 24 h timepoints). However, expression of the same genes showed minimal modulation or even upregulation in EP tumours that had acquired resistance to RMC-7977. These results were complemented by IHC staining for the protein product of *Birc5* (survivin) in the same tumours (Extended Data Fig. 6e,f), indicating that the acquisition of resistance to RMC-7977 coincided with the development of RAS–MAPK-independent expression and activity of the YAP pathway.

**Extended Data Fig. 7:**
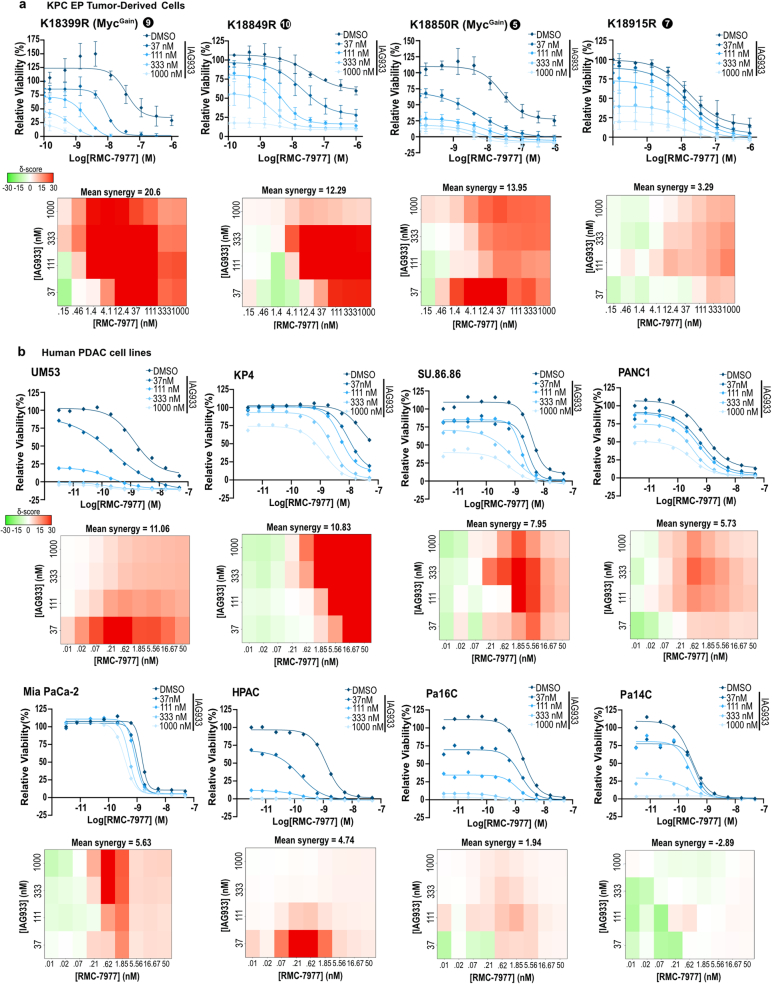
**(a)** Cell lines derived from RMC-7977 resistant KPC tumors and **(b)** human PDAC cell lines treated with DMSO, RMC-7977, IAG933 or combinations. (Top rows) Viability levels of cell lines treated with a range of RMC-7977 concentrations in combination with indicated concentration of IAG933. Line colors correspond to IAG933 concentration. Data points in (a) represent the mean of 3-4 biological replicates normalized to DMSO control. Error bars indicate ±s.d. (Bottom rows) The dose–response matrices show combination synergy based on cell viability at different dose pairs. Each cell line in (b) represents one biological replicate (n = 8). Source Data

To confirm pharmacologically that RMC-7977-resistant tumour cells relied on YAP pathway activity, we examined the response of KPC-derived cell lines to the pan-TEAD inhibitor IAG933^38^, either alone or in combination with RMC-7977. Western blot analysis found that levels YAP targets such as survivin, ECT2 and MYC protein were clearly inhibited in two RMC-7977 naive lines (although CYR61 protein was not), whereas MYC was only partially inhibited and other YAP targets were unchanged by RMC-7977 treatment in two resistant lines (Fig. 6e). Notably, the addition of IAG933 in combination with RMC-7977 fully inhibited levels of MYC and all other YAP targets examined in the resistant lines, and induced apoptosis as measured by cPARP in all cases. Finally, dose-escalation experiments using RMC-7977 in combination with IAG933 found that these agents have additive or synergistic effects in PDAC cell lines from both RMC-7977-resistant and naive KPC tumours (Fig. 6f and Extended Data Fig. 7a) as well as in seven out of eight human PDAC cell lines (Extended Data Fig. 7b). Together, these results suggest a potential means by which to counteract resistance to multi-selective RAS(ON) inhibition.

The possibility of a mutation-agnostic RAS inhibitor as a therapeutic agent for RAS-addicted cancers has been entangled with the widely held assumption that targeting wild-type RAS in humans would prove intolerable. Indeed, there is little evidence from mouse models to guide expectations for the effects of widespread inhibition of canonical RAS family members. Homozygous knockout of *Kras* produces an early (e3.5) embryonic lethal phenotype^20^, and conditional deletion in haematopoietic lineages eventually compromises haematopoiesis^21^. However, neither of these experiments accurately model the inhibition of RAS in humans that could be achieved with a small molecule, broad-spectrum RAS inhibitor. Perhaps the closest parallel is the systemic inhibition of MYC (which serves as a conduit for RAS signalling in many cell types) through inducible expression of the dominant negative protein Omomyc^39^. This approach showed that inhibition of physiological MYC activity reduced proliferation in most epithelial tissues, but that key epithelial functions were broadly maintained for extended periods of time. Systemic RAS inhibition may prove to be similar in nature, with tolerability enabled by the relatively low levels of active RAS-GTP (the target for RMC-7977) in normal tissues^40^ and the reduced affinity of RMC-7977 for wild-type RAS compared with mutant variants^3^. This is consistent with prior work showing that unlike RAS-addicted tumour cells, normal cells can rapidly restore homeostasis following RAS pathway inhibition^41^. The distinct anti-proliferative, pro-apoptotic effects of RMC-7977 in KRAS-mutant tumour cells relative to normal tissues are consistent with the concept of oncogene dependence and explain the remarkable extension of overall survival that we observed in the highly chemoresistant KPC mouse model.

As RAS(ON) multi-selective inhibitors such as RMC-6236 progress through clinical development, the critical questions of response duration and mechanisms of resistance will become central. PDAC is a remarkably plastic malignancy that is capable of adapting to and overcoming extreme environments and aggressive interventions. However, aberrant RAS signalling is the fundamental pillar on which PDAC biology is built; the clinical experience with approved RAS(G12C) inhibitors, particularly in non-small cell lung cancer, indicates that restoration of mitogenic RAS signalling is a frequent and preferred resistance mechanism in tumours if given the opportunity. Our evidence suggests that targeting both mutant and wild-type RAS proteins makes the path to resistance steeper for pancreatic tumours, largely precluding some of the mechanisms that are commonly observed with mutation-selective RAS pathway inhibitors. Ongoing clinical studies with RMC-6236 may reveal whether such a restricted range of resistance mechanisms directly translates to more durable responses in humans. Should MYC alterations prove common in patients whose tumours progress on treatment RAS(ON) multi-selective inhibition, combined targeting of the RAS and YAP–TAZ–TEAD pathways may provide an avenue of investigation for treating PDAC tumours with such alterations. Together, our findings have important and positive implications for the translation of RAS(ON) multi-selective inhibitors, as exemplified by RMC-6236 monotherapy, in patients with PDAC and potentially other types of RAS-addicted cancers.

## Methods

### RMC-7977 formulation

For in vitro studies RMC-7977 was re-suspended in DMSO (Fisher Bioreagents, BP231-100) and used at 10 mM stock concentration. For use in the in vivo studies RMC-7977 was prepared using the formulation made of 10/20/10/60 (%v/v/v/v) DMSO/PEG 400/Solutol HS15/water. The same vehicle formulation was used for all control groups.

### Cell culture and reagents

PDX human PDAC cell lines were provided by A. Maitra: Pa01C, Pa02C, Pa14C, and Pa16C. hF39 and hF43 cell lines were provided by D. Tuveson. The UM147 PDX cell line was obtained from University of Michigan (PMID: 17283135). PaCaDD-137 and PaCaDD-165 were obtained from the German Collection of Microorganisms and Cell Cultures GmbH (DSMZ; https://www.dsmz.de/). All remaining cell lines were obtained from the American Type Culture Collection (ATCC). Cell lines were grown in appropriate medium supplemented with 1% penicillin-streptomycin and fetal bovine serum (FBS) at 15% for UM147 or 10% for all other cell lines and maintained at 37 °C in a humidified incubator at 5% CO_2_, unless otherwise indicated. PaCaDD-137 and PaCaDD-165 were cultured in 80% mixture of DMEM and defined keratinocyte serum-free medium (at 1:1 ratio) supplemented with penicillin-streptomycin at 1% and FBS at 20%.

Mouse PDAC cell lines were derived from tumour-bearing KPCY (6419c5 and 2838c3^42^) or KPC (4662-G12D^43^) mice on a congenic C57BL/6 background. The 4662-G12C line was generated using CRISPR–Cas9 to replace the endogenous G12D mutation from 4662-G12D cells with the G12C mutation by lentiviral transduction. *Kras* allele states were confirmed by genomic sequencing. Mouse RMC-7977-resistant cell lines were isolated from KPC mice treated with RMC-7977 until endpoint. Cells were cultured in Dulbecco’s modified Eagle medium (DMEM, high glucose without sodium pyruvate) supplemented with 10% heat-inactivated FBS and 1% penicillin-streptomycin. RMC-7977 at 10 nM was added to the resistant cell lines, medium was changed every other day with inhibitor freshly added each time. Cell line information is provided in Supplementary Table 5.

### PRISM assay

The PRISM dataset generation and analysis are described in the accompanying Article^3^.

#### Cell lines

The PRISM cell set consisted of 796 cell lines representing more than 45 lineages cell line information in Supplementary Table 6, which largely overlapped with the Cancer Cell Line Encyclopedia (CCLE; https://portals.broadinstitute.org/ccle). Cell lines were grown in RPMI without phenol red, supplemented with 10% or 20% FBS for adherent and suspended lines, respectively. Parental cell lines were stably infected with a unique 24-nucleotide DNA barcode via lentiviral transduction and blasticidin selection. After selection, barcoded cell lines were expanded and subjected to quality control (mycoplasma contamination test, a SNP test for confirming cell line identity, and barcode ID confirmation). Approved cell lines were then pooled (20–25 cell lines per pool) based on doubling time similarity and frozen in assay-ready vials.

#### PRISM screening

RMC-7977 was added to 384-well plates at 8-point concentration with threefold dilutions in triplicate. These assay-ready plates were then seeded with the thawed cell line pools. Adherent cell pools were plated at 1,250 cells per well, while suspension and mixed adherent or suspension pools were plated at 2,000 cells per well. Treated cells were incubated for 5 days, then lysed. Lysate plates were collapsed together prior to barcode amplification and detection.

#### Barcode amplification and detection

The unique barcode for each cell line is located in the 3′ untranslated region of the blasticidin-resistance gene and is therefore expressed as mRNA. Total mRNA was captured using magnetic particles that recognize polyA sequences. Captured mRNA was reverse-transcribed into cDNA and then the sequence containing the unique PRISM barcode was amplified using PCR. Finally, Luminex beads that recognize the specific barcode sequences in the cell set were hybridized to the PCR products and detected using a Luminex scanner which reports signal as a median fluorescent intensity (MFI).

#### Data processing

1. Each detection well contained ten control barcodes in increasing abundances as spike-in controls. For each plate, we first create a reference profile by calculating the median of the log_2_(MFI) values across negative control wells for each of these spiked-in barcodes.
2. For each well, a monotonic smooth p-spline was fit to map the spike in control levels to the reference profile. Next, we transform the log_2_(MFI) for each cell barcode using the fitted spline to allow well-to-well comparisons by correcting for amplification and detection artifacts.
3. Next, the separability between negative and positive control treatments was assessed. In particular, we calculated the error rate of the optimum simple threshold classifier between the control samples for each cell line and plate combination. Error rate is a measure of overlap of the two control sets and was defined as Error = (FP + FN)/*n*, where FP is the number of false positives, FN is the number of false negatives, and *n* is the total number of controls. A threshold was set between the distributions of positive and negative control log_2_(MFI) values (with everything below the threshold said to be positive and above said to be negative) such that this value is minimized. Additionally, we also calculated the dynamic range of each cell line. Dynamic range was defined as DR = *μ*_−_ − *μ*_+_, where *μ*_+_ and *μ*_−_ are the median of the normalized log_2_(MFI) values in positive and negative control samples, respectively.
4. We filtered out cell lines with error rate above 0.05 or a dynamic range less than 1.74 from the downstream analysis. Additionally, any cell line that had fewer than two passing replicates was also omitted for the sake of reproducibility. Finally, we computed viability by normalizing with respect to the median negative control for each plate. Log-fold-change viabilities were computed as log-viability = log_2_(*x*) – log_2_(*μ*_−_), where log_2_(*x*) is the corrected log_2_(MFI) value in the treatment and log_2_(*μ*_−_) is the median corrected log_2_(MFI) in the negative control wells in the same plate.
5. log-viability scores were corrected for batch effects coming from pools and culture conditions using the ComBat algorithm^44^.
6. We fit a robust four-parameter logistic curve to the response of each cell line to the compound: $f(x)=b+(a-b)/(1+{{\rm{e}}}^{(s\log (x/{{\rm{EC}}}_{50}))})$. We used the following restrictions: (i) We require that the upper asymptote of the curve be between 0.99 and 1.01; (ii) we require that the lower asymptote of the curve be between 0 and 1.01; (iii) we do not enforce decreasing curves; (iv) we initialize the curve fitting algorithm to guess an upper asymptote of 1 and a lower asymptote of 0.5; and (v) when the standard curve fit fails, we report the robust fits provided by the dr4pl R package. We then computed AUC values for each dose–response curve and IC_50_ values for curves that dropped below 50% viability.

Finally, the replicates were collapsed to a treatment-level profile by computing the median log-viability score for each cell line.

#### Associations between inhibitor sensitivity AUC and mutations

For every gene with non-silent mutations in at least four cell lines, we compared the AUC values between cells with and without those mutations using a *t*-test. This analysis was carried out for: (1) the full dataset; (2) excluding cell lines with non-silent *KRAS* mutations; and (3) excluding cell lines that have either *KRAS* or *NRAS* non-silent mutations.

#### Bioinformatics analyses

Gene mutation, gene expression and lineage data were downloaded from the 22Q4 release of the DepMap Data Portal^45^. For tumour models with no publicly available data, we carried out whole-exome sequencing to ascertain gene mutations and RNA sequencing to ascertain gene expression. DNA mutation calling was accomplished with TNSeq using the hg38 version of the human genome^46^. Functional annotation of the resulting mutation calls was accomplished with Variant Effect Predictor and further annotated with oncoKB^47^. Gene expression was quantified using salmon against the hg38 version of human transcriptome further processed using txImport and edgeR to generate normalized counts^48–50^.

### Mouse cell viability assays

PDAC mouse cell lines with *Kras*^G12C^ or *Kras*^G12D^ mutations (treatment-naive or derived from RMC-7977-treated endpoint KPC tumours) were seeded at 2 × 10^3^ in a 96-well plate. Cells were treated 24 h later with DMSO or serial dilutions of RMC-7977, ERK inhibitor (SCH772984) or MEK inhibitor (trametinib). Cell viability was evaluated 72 h later by measuring ATP levels using the CellTiter-Glo Luminescent Cell Viability Assay (Promega, G7572) according to the manufacturer’s instructions. Alternatively (in experiments comparing naive and resistant cell lines), live cells were fluorescently labelled using Calcein AM (20-min incubation at 500 nM, Thermo Fisher) and counted using the SpectraMax i3X multimode detection platform (Molecular Devices). Technical triplicates were run for each biological replicate and a total of 3–4 biological replicates was done for each cell line. Growth percentage was calculated by normalizing drug-treated values to DMSO control, which was set to 100%. Four-parameter drug response curves were generated from biological replicates in GraphPad Prism. Mean ± s.d. was plotted for each tested dilution.

For synergy evaluation testing RMC-7977 treatment-naive and resistant cell lines, a similar protocol was used with following change: 24 h post cell line seeding, RMC-7977, IAG933 (Nantong Hi-future Biotechnology, 2714434-21-4), or combined treatment were added to the cells using the D300e digital dispenser (Tecan). Mean synergy value for each cell line was calculated using Excess over Bliss method, using SynergyFinder package in R Studio.

### Human cell line proliferation assay

19 PDAC cell lines were tested for sensitivity to RMC-7977 as part of a panel of human cancer cell lines of various histotypes screened at Crown Bioscience. These PDAC cell lines harboured *KRAS*^G12D^, *KRAS*^G12V^, *KRAS*^G12C^, *KRAS*^Q61H^ and *BRAF*^V487_P492delinsA^ mutations. To measure inhibition of cell proliferation, cells were cultured in methylcellulose and treated in triplicates with serial dilutions of RMC-7977 (top concentration of 1 µM) or DMSO dispensed by a Tecan D300e digital dispenser (Tecan). Cells were incubated for 120 h prior to measurement of ATP levels using CellTiter-Glo. Technical triplicates were run for each biological replicate and a total of 3–4 biological replicates were done for each cell line. Growth percentage was calculated by normalizing drug-treated values to DMSO control, which was set to 100%. Normalized CTG assay readouts were plotted as a function of log molar inhibitor concentration and a four-parameter sigmoidal concentration–response model was fitted to the data. Mean ± s.d. was plotted for each tested dilution.

PDAC cell lines harbouring wild-type *KRAS* or *KRAS*^Q61H^ were plated at 500–4,000 cells per well in clear, flat-bottomed 96-well plates (Corning) and grown for 24 h prior to adding indicated concentration of RMC-7977 or DMSO using the D300e digital dispenser (Tecan). Following treatment, cells were incubated for additional 3–5 days after which live cells were fluorescently labelled using Calcein AM (20-min incubation at 500 nM, Thermo Fisher) and counted using the SpectraMax i3X multimode detection platform (Molecular Devices). Experiments were day 0 normalized using an independent culture plate. Growth percentage was calculated by normalizing drug-treated values to DMSO control, which was set to 100%. Four-parameter sigmoidal concentration–response models were fitted to the data from at least three biological replicates. Mean ± s.d. was plotted for each tested dilution.

For synergy evaluation of combined RMC-7977 and IAG933, a similar protocol was used with following change: 24 h post cell line seeding, RMC-7977, IAG933 (Nantong Hi-future Biotechnology, 2714434-21-4), or the combinations were added to the cells using the D300e digital dispenser (Tecan). Each cell line was considered as a separate biological replicate (*n* = 8). Mean synergy value for each cell line was calculated using the excess over bliss method, using SynergyFinder package in R(Studio).

### Western blot analysis

Cells were seeded at 7.5 × 10^3^ to 4 × 10^6^ cells per well in 6-well plates or 100-mm dishes in growth medium. After overnight incubation, indicated compound (RMC-7977, IAG933 or DMSO (0.1% v/v)) were added and incubated for the indicated timepoints. Cells were washed twice with ice-cold PBS and lysed with NP-40 lysis buffer (Thermo Fisher, J60766), MSD Tris Lysis Buffer (MSD, R60TX-2), RIPA buffer (50 mM TRIS-HCl, pH 7.5, 150 mM NaCl, 1% NP-40, 0.5% sodium deoxycholate, 0.1% SDS) or a lysis buffer containing 1% Triton X-100, 20 mM Tris-HCl, 150 mM NaCl, and 1 mM EDTA. All lysis buffers were supplemented with protease and phosphatase inhibitors. Lysates were scraped and collected before centrifugation at 21,000*g* for 10 min at 4 °C. The protein-containing supernatants were quantified by BCA assay (Pierce, 23225) and equal quantities of protein were denatured with LDS and reducing agent at 95 °C. Samples were resolved on 12% or 4–12% Bis-Tris polyacrylamide gels, then transferred to a nitrocellulose or PVDF membrane using the iBlot 2.0 system or wet transfer. Membranes were blocked in Intercept TBS buffer (Li-Cor, 927-60001) or 3-5% milk before probing with primary antibodies overnight at 4 °C. Secondary antibodies were added as appropriate, and the membranes were imaged on a Li-Cor Odyssey imager. Alternatively, membranes were incubated with HRP-linked secondary antibodies and developed with Clarity or ClarityMax chemiluminescent substrates using a ChemiDoc XRS+ or ChemiDoc MP imager (Bio-Rad).

The following primary antibodies were used at 1:1,000 dilution for western blot analysis: anti-phospho-p44/42 MAPK (ERK1/2) T202/Y204 (9101, 4370), anti-p44/42 (ERK1/2) (9107, 4695; 9102; 4696), anti-PARP (9532), anti-pAKT (Ser473) (9271), anti-AKT (40D4) (2920), anti-AKT (Thr308) (244F9) (4056), anti-pS6 (Ser235/236) (2211), anti-S6 (54D2) (2317), anti-MYC (D84C12) (5605), anti-survivin (2808), anti-CDC20 (14866), anti-CYR61 (39382) and anti-vinculin (13901) from Cell Signaling Technology; anti-vinculin (V9131) from Sigma; anti-ECT2 (07-1364) from Millipore. Anti-alpha-tubulin (3873) from CST and anti-beta-tubulin (66240-1-1g) from Proteintech were used at 1:2,000 dilution. Anti-cPARP (9541) from CST was used at 1:750 dilution. The following secondary antibodies were used according to manufacturer’s recommendation: goat anti-rabbit IR800-conjugated (926-32211), goat anti-mouse IR680-conjugated (926-68070), goat anti-mouse IR800-conjugated (926-32210) from Li-COR; HRP-linked anti-rabbit (7074) and HRP-linked anti-mouse (7076) from CST; IgG (H+L) cross-adsorbed goat anti-mouse HRP (PI31432) and IgG (H+L) cross-adsorbed goat anti-rabbit HRP (PI31462) from Invitrogen, goat anti-mouse IgG (H+L) cross-adsorbed secondary antibody HRP (31432), goat anti-rabbit IgG (H+L) cross-adsorbed secondary antibody HRP (31462) from Thermo Fisher. Antibody information is provided in Supplementary Table 7.

### PDAC organoid preparation and treatment conditions

#### Origins and genetic profiling of patient-derived organoids

Genetic profiling was performed on tissue biopsies from patients using whole-genome sequencing or OncoPanel^43,51^. All patients consented to an Institutional Review Board (IRB)-approved protocol at Dana-Farber Cancer Institute permitting access to their clinical and genomic data.

#### Organoid culture

Organoids were cultured at 37 °C in 5% CO_2_. Cells were seeded in growth factor reduced Matrigel (Corning; 356231) domes and incubated with human organoid medium formulated as follows: Advanced DMEM/F12-based-conditioned medium, 1× B27 supplement, 10 mM HEPES, 2 mM GlutaMAX, 10 mM nicotinamide, 1.25 mM *N*-acetylcysteine, 50 ng ml^−1^ mouse EGF, 100 ng ml^−1^ human FGF10, 0.01 μM human gastrin I, 500 nM A83–01, 100 ng ml^−1^ noggin, 1× WNT3A conditioned 10% FBS DMEM (50% by volume) and 1× R-spondin conditioned basal medium (10% by volume)^52,53^.

#### Organoid drug treatment and viability assay

Organoids were dissociated using TrypLE Express (Thermo Fisher, 12604054) and cells were seeded into ultra-low attachment 384-well plates at 1 × 10^3^ cells per well into 20 μl of culture media, consisting of 10% Matrigel and 90% human organoid medium. Organoids were treated 24 h post seeding over a 12-point dose curve with RMC-7977 or with DMSO in a randomized fashion using a Tecan D300e Digital Dispenser. Cell viability was assessed 6 days post-treatment using a Cell-TiterGlo 3D Cell Viability assay (Promega, G9683), according to the manufacturer’s instructions. Fluorescence was read using a FLUOstar Omega microplate reader. Technical triplicates were analysed for each biological replicate and a total of three biological replicates were done for each cell line. Growth percentage was calculated by normalizing drug-treated values to DMSO control, which was set to 100%. CTG assay readouts were plotted as a function of log molar [inhibitor] and a 4-parameter sigmoidal concentration–response model was fitted to the data. Mean ± s.d. was plotted for each dilution.

### Ex vivo human PDAC explant preparation and treatment conditions

Explant culture sponges and optimized culture media were prepared as previously described^25^. Human tissue samples were obtained from de-identified patients undergoing resection surgeries, primarily pancreaticoduodenectomy (Whipple) or distal pancreatectomy, at New York Presbyterian/Columbia University Irving Medical Center. Upon receipt of a resected human PDAC fragment (*n* = 4), each tissue sample was cut into 300 μm slices using a Compresstome. Any tumour tissue remaining after sectioning was fixed in 4% paraformaldehyde (Santa Cruz Biotechnology, sc-281692) for 2 h, at 4 °C as the Day 0 control. Sectioned slices were next placed between gelatin sponges pre-soaked in 750 µl of media containing either DMSO or varying concentrations of RMC-7977 (10–100 nM). Each well of a 24-well plate contained one explant slice placed between bottom sponge (1 cm^3^) and a top sponge (2–3 mm thick). After 24 h culture, explants were collected and fixed in 4% PFA for 2 h. Fixed tissue was then transferred to 70% ethanol and paraffin embedded for long-term storage and further analysis.

### In vivo xenograft studies

#### Mouse studies

Studies were conducted at the following CROs: GenenDesign (Shanghai, China), Pharmaron (Beijing, China) and Wuxi AppTec (SuZhou, China). All CDX/PDX mouse studies and procedures related to animal handling, care and treatment were conducted in compliance with all applicable regulations and guidelines of the relevant Institutional Animal Care and Use Committee (IACUC). Female BALB/c nude mice and NOD SCID mice 6–8 weeks old from Beijing Vital River/VR Laboratory Animal Co., Beijing AniKeeper Biotech and Shanghai Sino-British SIPPR/BK Laboratory Animal Co. were used for these studies.

#### Generation of xenograft models

In order to generate subcutaneous xenograft tumours each mouse was inoculated at the right flank with tumour cells (2 × 10^6^ − 1 × 10^7^) in 100–200 µl of medium/PBS supplemented with Matrigel (1:1). Treatments were started when the average tumour volume reached 150–250 mm^3^ (for tumour growth evaluation) and 400–600 mm^3^ (for single dose pharmacokinetic–pharmacodynamic study). Tumour diameter was measured in two dimensions using a digital calliper, and the tumour volume in mm^3^ was calculated using the formula: volume = (width^2^ × length)/2. Mice on studies were weighed and tumours were measured two times a week.

The human primary cancer xenograft models were generated using fresh tumour fragments obtained from hospitals with informed consent from the patients in accordance with protocols approved by the Hospital Institutional Ethical Committee (IEC). The tumour fragments were serial passaged in BABL/c nude mice and then cryopreserved for further use. For this study, recovered tumour fragments of about 15–30 mm^3^ in size from each model were implanted into right flanks of BALB/c nude mice. Treatment started when average tumour volume reached 150–250 mm^3^.

To generate orthotopic xenograft tumours, survival surgeries were carried out and 2 × 10^6^ to 5 × 10^6^ luciferase-expressing tumour cells in 30–50 µl media/Matrigel mixtures (1:1) were implanted directly into the mouse pancreas. Treatments were started when the tumours produced an average of 50–80 × 10^7^ photons s^−1^ as measured by the in vivo imaging system (IVIS). All subsequent tumour measures were also conducted by IVIS. For routine monitoring, mice were injected intraperitoneally with 15 mg ml^−1^ (at 5 µl g^−1^ BW) of d-Luciferin (Perkin Elmer) and imaged, after which Living Image software (Perkin Elmer) was used to compute regions of interest (ROI) and tumour volumes.

#### RMC-7977 treatment

Tumour-bearing mice were randomized and assigned into groups (*n* = 3–10 per group). Vehicle or RMC-7977 was administered via oral gavage daily at 10 mg kg^−1^ and mice were treated for 21–28 days. Studies were terminated early if tumour burden reached humane endpoint. Body weights were collected twice a week during the study. Means ± s.e.m were plotted in the waterfall plots. For the single-dose pharmacokinetic–pharmacodynamic study, mice were randomized and assigned into groups (*n* = 3–6 per dose and timepoint). A single dose of RMC-7977 was administered orally at 10 mg kg^−1^, 25 mg kg^−1^ or 50 mg kg^−1^. Tissues (including tumour, colon and skin) were collected at indicated timepoints and either fixed in 10% formalin, embedded in Optimal Cutting Temperature (OCT; Sakura, 4583) solution or snap-frozen in liquid nitrogen for further analysis. Whole blood was transferred into K_2_EDTA Microtainer tubes (BD, 365974), incubated for 5 min and snap-frozen in liquid nitrogen.

### In vivo allograft studies

#### Mouse studies

All mouse allograft studies and procedures related to animal handling, care and treatment were conducted in compliance with all applicable regulations and guidelines of the Institutional Animal Care and Use Committee (IACUC). Female C57BL/6J (strain 000664) mice aged 6–8 weeks from the Jackson Laboratory were used for these studies.

#### Generation of allograft models

In order to generate subcutaneous allograft tumours, each mouse was inoculated in the right flank with 3 × 10^5^ of KPCY 6499c4 tumour cells in 0.1 ml of Matrigel:PBS (1:1). Treatments were started when the average tumour size reached 140 mm^3^. Tumour size was measured at two dimensions using a digital calliper, and the tumour volume in mm^3^ was calculated using the formula volume = (width^2^ × length)/2. Mice on studies were weighed and tumours were measured 2 times a week.

To generate orthotopic allograft tumours, 5 × 10^4^ KPCY 6499c4 tumour cells in 20 µl PBS/Matrigel mixtures (1:1) were implanted directly into the mouse pancreas through a laparoscopic incision. Treatments were started when the average tumour size reached ~50 mm^3^. Body weights were measured and tumour growth was monitored by ultrasound twice weekly.

#### RMC-7977 treatment

Tumour-bearing mice were randomized, assigned into groups (*n* = 9–10 per group), and treated daily via oral gavage with vehicle or RMC-7977 (10 mg kg^−1^). For subcutaneous KPCY study, survival endpoint was defined as: tumour volume reaching 2000 mm^3^ or mice showing any clinical signs, including severe ulceration. For orthotopic KPCY study, survival endpoint was defined as (1) mice showing any clinical signs including hunching or fluid in the abdomen, or (2) tumour dimensions exceeding the imaging frame of the ultrasound. Body weights were measured twice a week during the study. Tissue was collected either at 4 h or 24 h after last dose and preserved as previously described (see ‘In vivo xenograft studies’).

### In vivo GEMM studies

#### Mouse breeding

All animal research experiments were approved by the Columbia University Irving Medical Center (CUIMC) Institutional Animal Care and Use Committee (IACUC). Mouse colonies were bred and maintained with standard mouse chow and water, ad libitum, under a standard 12 h light/12 h dark cycle. KPC (*Kras*^*LSL.G12D/+*^; *Trp53*^*LSL.R172H/+*^;*Pdx1-cre*^*tg/+*^), KC (*Kras*^*LSL.G12D/+*^;*Pdx1-cre*^*tg/+*^), PC (*Trp53*^*LSL.R172H/+*^;*Pdx1-cre*^*tg/+*^) as well as KP^F/F^C (*Kras*^*LSL.G12D/+*^;*Trp53*^*flox/flox*^;*Pdx1-cre*^*tg/+*^), KP^F/F^ (*Kras*^*LSL.G12D/+*^;*Trp53*^*flox/flox*^) and P^F/F^C (*Trp53*^*flox/flox*^;*Pdx1-cre*^*tg/+*^) mice were generated in the Olive Laboratory at Columbia University, by crossing the described alleles. Mouse genotypes were determined using real-time PCR with specific probes designed for each gene (Transnetyx). *Kras*^LSL-G12D/+^;*Trp53*^*LSL-R172H/+*^;*Pdx1-cre*^*tg/+*^;*Rosa26*^YFP/YFP^ (KPCY) were bred and maintained in pathogen-free facilities at the University of Pennsylvania.

#### Pharmacokinetic–pharmacodynamic study in KP^F/F^C

Tumour formation in KP^F/F^C mice was monitored by bi-weekly palpations until the detection of a mass, which was then confirmed by ultrasound. Tumour-bearing mice were randomized and assigned into groups (*n* = 3 per dose and timepoint). Single dose of vehicle or RMC-7977 was administered orally at 10 mg kg^−1^, 25 mg kg^−1^ or 50 mg kg^−1^. Whole blood and tissue (tumours and colons) were collected at indicated timepoints and preserved as previously described (see ‘In vivo xenograft studies’).

#### Pharmacodynamic study in KPC mice

Tumour formation in KPC mice was monitored by bi-weekly palpation. Upon detection of a 4–7 mm diameter tumour by ultrasound, KPC mice were randomized and treated with vehicle (*n* = 6) or RMC-7977 (50 mg kg^−1^; *n* = 11). Treatments were performed every other day via oral gavage for 1 week. Mouse health status and weight were checked daily and ultrasounds (Vevo 3100) were performed every third day to monitor tumour growth. Following two consecutive ultrasounds, RMC-7977-treated mice were euthanized either 4 (*n* = 7) or 24 h (*n* = 4) after last dose and vehicle-treated mice were euthanized between 4–24 h post last dose. Tissue was collected and preserved as previously described (see ‘In vivo xenograft studies’). Additional group of KPC mice was also treated with a single dose of RMC-7977 (*n* = 10) or vehicle (*n* = 3) and tissues were collected at 4 or 24 h post-dose as previously described.

#### Pharmacodynamic study in KPCY mice

KPCY mice were enroled upon detection of a 15–100 mm^3^ tumour measured via ultrasound. Mice were randomized into groups and treated with vehicle (*n* = 6) or RMC-7977 (25 mg kg^−1^; *n* = 8). Treatments were performed every day via oral gavage for 15 days and ultrasounds were performed on day 8 and 15. Mice were euthanized after last dose and tissue was collected and preserved as previously described (see ‘In vivo xenograft studies’).

#### Survival study in KPC mice

For survival study, KPC mice with 4–7 mm diameter tumours (as measured by ultrasound) were enroled and treated every other day with vehicle (*n* = 9) or RMC-7977 (50 mg kg^−1^; *n* = 13). Mouse health status and weight were checked daily and ultrasounds were performed every third day to monitor tumour growth. The survival endpoint was determined by overall health criteria scoring, where endpoint is determined by a score of 5 or greater based on the following criteria: moribund, immediate euthanasia; abdominal distention due to haemorrhagic ascites, 5 pts; mild difficulty beathing, 5 points; hypothermia, 5 points; abdominal distention due to chylous ascites, 3 points; loss of over 20% enrolment body weight, 3 points; failure of grasp test, 3 points; jaundice or pallor, 3 points; weak grasp test, 2 points; failure to interact with other mice, 1 point; hunched, 1 point; pilorection/failure to groom, 1 point.

Additional notes were made to better characterize the cause of death upon necropsy, including the presence of macro liver and/or lung metastases, jaundice, and tumour-mediated gastrointestinal obstructions. Survival is denoted as Kaplan–Meier survival curves compared with a log-rank, Mantel–Cox test. Mice that reached endpoint criteria were euthanized either at 4 or 24 h after last dose in a manner consistent with IACUC standards and our own criteria scoring. Tissue was collected at time of necropsy for further analysis.

### In vivo pharmacodynamic analysis by RT–qPCR

RNA was extracted from at least 20 mg of indicated OCT or liquid nitrogen frozen tissue using an RNeasy Mini Kit (Qiagen, 74104) and a high-throughput tissue grinder following the manufacturer’s protocol. Reverse transcription was carried out using High-Capacity cDNA Reverse Transcription Kit (ABI, 4368814) according to the manufacturer’s protocol. The cDNA product was used for quantitative PCR analysis using TaqMan Gene Expression Master Mix (ABI, 4369016) or iTaq Universal SYBR Green Supermix (Bio-Rad, 172-5125) depending on the primer. TaqMan primer probes specific to *Dusp6* (and human *DUSP6*), *Yap1*, *Cyr61*, *Ankrd1*, *Amotl2*, *Ect2*, *Birc5* and 18S (mouse and human, used as an internal control gene) were used to detect gene levels in each sample in duplicates or triplicates using a 10 µl final reaction volume in a 96 or 384-well plate. Standard primer sequences specific for *18S*, *Ccnd1*, *Epha2*, *Etv4* and *Spry2* were used to detect the gene levels in each sample in duplicates or triplicates using a 10 µl final reaction volume in a 96-well plate. For RT–qPCR analysis, *C*_t_ values were normalized to 18S RNA, and then the mean mRNA expression levels of each sample were normalized to the average of the vehicle control group. Values were plotted as fold change in mRNA expression compared to vehicle. Means ± s.d. were shown. Primer sequences and further information are provided in Supplementary Tables 9 and 10.

### Mouse blood and tissue sample bioanalysis

Whole blood, tumour, colon and skin tissue concentrations of RMC-7977 were determined using liquid chromatography–tandem mass spectrometry (LC–MS/MS) methods. Tissue samples were homogenized with a 5× or 10× volume of homogenization buffer (methanol/15 mM PBS (1:2; v:v) or 15 mM PBS with 10% methanol). An aliquot of whole blood or homogenized tissue (10 or 20 µl) was transferred to 96-well plates (or tubes) and quenched with a 20× volume of acetonitrile/methanol (1:1; v/v) with 0.1% formic acid containing a cocktail of internal standards. After thorough mixing and centrifugation, the supernatant was directly analysed on a Sciex 6500+ triple quadrupole mass spectrometer equipped with an ACQUITY or Shimadzu UPLC system. An ACQUITY UPLC BEH C18 or C4 1.7 μm (2.1 × 50 mm) column was used with gradient elution for compound separation. RMC-7977 and internal standard (verapamil or terfenadine) were detected by positive electrospray ionization using multiple reaction monitoring (RMC-7977: *m*/*z* 865.4/706.4 or *m*/*z* 865.3/833.5; verapamil: *m*/*z* 455.2/164.9; terfenadine: *m*/*z* 472.3/436.4). The lower limit of quantification was 0.5 ng ml^−1^ or 2.0 ng ml^−1^ for blood, tumour, and other tissue. BA analysis on blood and tissue samples from xenograft models was run at Wuxi AppTec. BA analysis on blood and tissue samples from allograft models and GEMM was run at Revolution Medicines.

### Immunohistochemistry

All tissues were fixed for up to 24 h using 10% neutral buffered formalin and then moved to 70% ethanol for long-term storage. All stainings were performed on 4-µm tissue sections. Sections were deparaffinized using a Leica XL ST5010 autostainer, after which slides were subjected to heat-activated epitope retrieval (1× citrate pH 6 or 1× EDTA pH 8). To block endogenous peroxidases, 20-min incubation in 3% H_2_O_2_ (Fisher Scientific) was performed. Slides were further blocked in serum for 1 h, and primary antibodies were added for overnight incubation at 4 °C. The next day, slides were washed and incubated with ImmPRESS HRP Horse Anti-Rabbit IgG Polymer Detection Kit (Vector Laboratories, MP-7401) for 30 min. Following incubation, ImmPACT DAB peroxidase (Vector Laboratories, SK-4100) was used to develop the stain and haematoxylin was used as nuclear counterstain. Stained slides were imaged at 40× magnification. Quantitative analyses of IHC was performed using ImageJ.

To stain tissues collected from the Capan-1 xenograft model, a similar protocol was used with the following changes. Sections were stained using a Leica BOND automated staining system and primary antibodies were detected with the Leica BOND Polymer detection kit (3-P-PV6119). Stained slides were scanned and digitized with a 3DHistotech Pannoramic whole slide scanner at 20× magnification. Image analysis was performed using HALO software from Indica Labs.

To stain tissues collected from the KPCY allograft model, a Biocare IntelliPATH automation system was used, and primary antibodies were detected with the MACH4-HRP-polymer Detection System (Biocare, MRH534). Stained slides were scanned and digitized with a TissueScope LE (Huron Digital Pathology) whole slide scanner at 20× magnification. Image analysis was performed using HALO software from Indica Labs.

The primary antibodies used for IHC were: anti-phospho-p44/42 MAPK (ERK1/2) T202/Y204 (4370, 1:200 (GEMM and Allograft Models) or 1:1,000 (Xenograft Models)), anti-cleaved caspase-3 (Asp175) (9661, 1:200 (GEMM models) or 1:400 (Xenograft Models)), anti-cleaved PARP (94885; 1:200 for GEMM models) and anti-survivin (2808; 1:500 for GEMM models) from Cell Signaling Technology; anti- cleaved caspase-3 (CP229, 1:100 (Allograft Models)) from Biocare Medical, anti-cyclin A2 (181591, 1:500 (GEMM models)) from Abcam, anti-pS6 ribosomal protein (Ser235/236) (2211, 1:200 (GEMM models)) from CST, anti-pS6 ribosomal protein (Ser240/244) (D68F8) XP (5364, 1:2000) from CST. Primary antibodies were detected using: ImmPRESS HRP Horse Anti-Rabbit IgG Polymer Detection Kit (MP-7401) from Vector Laboratories, MACH4 HRP-polymer Detection System (M4U534) from Biocare Medical, and Leica BOND Polymer detection kit (3-P-PV6119) from Leica Microsystems. Antibody information is provided in Supplementary Table 7.

### Dual Immunofluorescence

Manual staining steps were as follows. Slides were backed at 60 °C for 20 min, dewaxed, followed by heat induced epitope retrieval using Biocare DIVA Decloaker pH 6.2 at 95 °C for 20 min. Sections were then blocked using BioCare Peroxidase block for 10 min at room temperature and incubated with primary antibodies (anti-pS6 ribosomal protein (Ser235/236) (D57.2.2E, 4858, 1:100 dilution, CST) or anti-phospho-p44/42 MAPK (ERK1/2) T202/Y204 (4370, 1:100 dilution, CST)).

for 45 min at room temperature. After washing, slides were incubated with Biocare Mach4 Polymer-HRP for 30 min at room temperature before adding Opal 690 at 1:100 in Opal diluent buffer for 10 min at room temperature. After washing slides were treated with BioCare DIVA pH 6.2 elution buffer for 20 min at 95 °C and allowed to cool to room temperature for 20 min. After washing slides were incubated with CK19 (anti-keratin 19) (D7F7W, 13092, 1:200 dilution) for 45 min at 1:200 dilution at room temperature followed by incubation with Biocare Mach4 Polymer-HRP for 30 min. After washing slides were then incubated with Opal 480 at 1:100 in Opal diluent buffer for 10 min at room temperature. Nuclear DAPI stain was conducted for 10 min at room temperature before mounting the coverslips onto the slides using Prolong Gold anti-fade aqueous mounting medium. Whole slide images were generated using a Huron scanner at 20× resolution. Antibody information is provided in Supplementary Table 7.

### Laser capture microdissection of malignant cells from tumour tissue

In order to enrich for the malignant epithelial cells, laser capture microdissection was performed as described^54^. In brief, 8-µm cuts of OCT-embedded tissue (human or mouse tumour) blocks were transferred to PEN membrane glass slides and stained with cresyl violet acetate according to manufacturer’s protocol. Laser capture microdissection was performed on a PALM MicroBeam microscope (Zeiss), collecting at least 1000 cells per sample. Genomic DNA was extracted and libraries were prepared using the QIAamp DNA Micro kit (Qiagen).

### Genetic profiling of human PDAC explant samples

In order to identify *KRAS* mutations in exons 2 and 3, PCR analysis was carried out followed by Sanger sequencing. In brief, pre-designed *KRAS* primers were purchased from Invitrogen. DNA isolated from tumour explants was amplified using the following designed primers containing the M13 tail for sequencing (Invitrogen, Hs00459263_CE): forward primer 5′- TGTAAAACGACGGCCAGTGAGTGAACATCATGGACCCTGACA-3′ and reverse: 5′-CAGGAAACAGCTATGACCTTAAGCGTCGATGGAGGAGTTTG-3′. PCR amplification was performed using Platinum Taq DNA Polymerase High Fidelity kit (Thermo Fisher, 11304011) and PCR products were purified with the QIAquick PCR purification Kit (Qiagen, 281106) according to the recommended protocol. Sequencing reactions were performed in reverse directions using the M13 forward primer, and electropherograms were reviewed manually to detect any genetic alteration.

### Sparse whole-genome and targeted locus sequencing of KPC tumours

DNA from microdissected KPC tumour tissue was subject to whole-genome amplification as described before^55^. TruSeq indexed Illumina sequencing libraries where then constructed from WGA DNA and subjected to sparse whole-genome sequencing at a depth of roughly 3 million sequencing reads, enough to enable copy number ascertainment at a bin resolution of ~100 kb. Sequencing libraries were also processed for targeted sequencing of the *Myc* locus at a coverage of ~100×. For sparse whole-genome data processing, sequencing reads were mapped to mouse genome build mm9 while skipping the first 50 base pairs containing inline barcoding sequences as well as degenerate oligonucleotide-primed PCR quasi-degenerate sequence. Further processing involved indexing and sorting of uniquely mapped reads as well as removal of PCR duplicates. Uniquely mapped sequencing reads were counted in genomic bins/intervals that were computed using a previously developed algorithm while partitioning the genome into 20,000 bins of ~100 kb in length as described before^56^. Read counts were subsequently corrected for GC content using LOWESS smoothing algorithm, normalized and segmented using circular binary segmentation^57,58^. Given that laser capture microdissection processing of tissue enriches for cancer cells but does not entirely remove contaminating stromal cells, copy number inference was done relative to the mean of the genome, and not on absolute copy number states. To call copy number gains, we conditioned each event to satisfy two criteria: focality in length as well as a low ratio value of above 1.5 normalized depth. For targeted *Myc* locus sequencing data, processing was done for copy number as well as single nucleotide variant detection. For copy number, FASTQ files are mapped to the target genome using the BWA mapper (bwa mem). The BAM files are then processed using the seqDNAcopy library^59^ to first get pairwise counts for the target sample and control samples (bam2counts) into bins of 100 bp. The data are then segmented using the seqDNAcopy seqsegment method.

For single nucleotide variant calling, the data processing pipeline for detecting variants in Illumina HiSeq data is as follows. First the FASTQ files are processed to remove any adapter sequences at the end of the reads using cutadapt (v1.6). The files are then mapped using the BWA mapper (bwa mem v0.7.12). After mapping the SAM files are sorted and read group tags are added using the PICARD tools. After sorting in coordinate order, the BAMs are processed with PICARD MarkDuplicates. The marked BAM files are then processed using the GATK toolkit (v 3.2) according to the best practices for tumour normal pairs. They are first realigned using ABRA (v 0.92) and then the base quality values are recalibrated with the BaseQRecalibrator. Somatic variants are then called in the processed BAMs using muTect (v1.1.7) for single nucleotide variant and the Haplotype caller from GATK with a custom post-processing script to call somatic indels.

### Proteomic data preparation and analysis

#### Proteomic sample preparation

K8484 or K18905 cells were treated with either 10 nM RMC-7977 or equivalent volume DMSO for 24 h in triplicate. Lysates were prepared in 400–450 μl of lysis buffer (8 M urea, 50 mM NH_4_HCO_3_ (pH 7.5), 1× protease inhibitor cocktails (Roche) and 1× phosphatase inhibitor cocktails I and II (Sigma Aldrich)). Lysates were sonicated six times on ice. Following sonication, samples were centrifuged for 10 min at 21,000*g* at 4 °C to pellet molecular debris. Cell lysates (1 mg per sample) were reduced with 5 mM DTT for 45 min at 37 °C, alkylated with 15 mM iodoacetamide for 30 min in the dark at room temperature, and protein was precipitated at a 1:10 ratio with cold methanol. Protein precipitates were recovered by centrifugation at 5,000 rpm at 4 °C for 45 min and reconstituted in 50 mM ammonium bicarbonate pH 8 to achieve a 0.5 mg ml^−1^ concentration. Samples were digested with LysC (Wako, 1:75 w/w) for 2 h at 37 °C, then digested with trypsin (Promega, 1:75 w/w) overnight at 37 °C. Digested peptide samples were acidified and desalted using desalting spin columns (Thermo). Eluates were dried via vacuum centrifugation. Peptide concentration was determined using Quantitative Colorimetric Peptide Assay (Pierce).

Four pooled samples were created from the twelve experimental samples and all were labelled with TMTpro 16plex reagents (Thermo Fisher). Each sample (200 µg) was reconstituted with 50 mM HEPES pH 8.5 and individually labelled with 500 µg of TMTpro reagent for 1 h at room temperature. Labelling efficiency was evaluated by LC–MS/MS analysis of a pooled sample from 1 µl of each sample. After confirming >98% efficiency, samples were quenched with 50% hydroxylamine to a final concentration of 0.4%. Labelled peptide samples were combined (1:1) and desalted using Thermo desalting spin column followed by being dried via vacuum centrifugation. The dried TMT-labelled sample was fractionated offline using high pH reversed phase HPLC (Agilent 1260) using an Agilent ZORBAX 300Extend-C18 column (3.5-µm, 4.6 × 250 mm) with mobile phase A containing 4.5 mM ammonium formate (pH 10) in 2% (vol/vol) LC–MS grade acetonitrile, and mobile phase B containing 4.5 mM ammonium formate (pH 10) in 90% (vol/vol) LC–MS grade acetonitrile. The 96 resulting fractions were then concatenated in a non-continuous manner into 24 fractions and 5% of each were aliquoted, dried down via vacuum centrifugation and stored at −80 °C until further analysis.

#### LC–MS/MS analysis

The proteome and phosphoproteome fractions were analysed by LC/MS/MS using a Thermo Ultimate 3000 nLC coupled to an Exploris480 mass spectrometer (Thermo Scientific). Samples were injected onto an Ion Opticks Aurora C18 column (75 μm internal diameter × 15 cm, 1.6 μm particle size) and separated over a 70- or 100-min method. The gradient for separation consisted of 5–42% mobile phase B at a 250 nl/min flow rate, where mobile phase A was 0.1% formic acid in water and mobile phase B consisted of 0.1% formic acid in 80% acetonitrile. The Exploris480 was operated in turboTMTpro mode with a cycle time of 3 s. Resolution for the precursor scan (*m*/*z* 375–1,400) was set to 60,000 with a automatic gain control (AGC) target set to standard and a maximum injection time set to auto. MS2 scans (30,000 resolution) consisted of higher collision dissociate set to 38; AGC target set to 300%; maximum injection time set to auto; isolation window of 0.7 Da; fixed first mass of 110 *m*/*z*.

#### Proteomics mass spectrometry search

All mass spectrometry raw files were jointly searched using MaxQuant^60^ (MQ) 2.4.3.0 Andromeda search engine^61^ using the UniProt Mouse Reference Proteome^62^ (21,864 sequences, accessed October 2023) and known contaminants included in MQ. The peptide length was set to 8–25 with a maximum mass of 4,600 Da. False Discovery Rate (FDR) for peptide identification was set at <0.01 with a minimum of one razor peptide. Peptide search was matched between runs with a match time window of 0.7 min. MS2 reporter ion for TMTpro 16plex (Thermo) was searched using manufacturers isotope correction factors with a reporter mass tolerance of 0.003 Da. Reporter ions were filtered by a minimum precursor intensity fraction (PIF) of 0.75. Oxidation and N-terminal acetylation were set as variable modifications, and carbamidomethyl was set as fixed modifications. Digestion was set to Trypsin/P with three maximum missed cleavages. Default orbitrap settings were used for spectrometer.

#### Proteomics differential expression and gene set enrichment analysis

Global proteomic differential expression analysis was performed in R (v4.3.1) using LIMMA (v3.56.2)^63^. Contaminants and reverse sequences were removed, and sample intensities were log_2_-transformed and median normalized. Sample quality was assessed by total intensity distributions, principal component analysis, and sample correlation analyses. Proteins were median centred by the median of control samples (DMSO) before performing differential expression analysis. Missing data were not imputed. Gene set enrichment analysis was performed on differential expression analysis results using msigdbr (v7.5.1) and fgsea (v1.26.0).

### Generation of experimental MAPK gene expression signature

#### Drug perturbation assays and PLATE-seq experiment

The PLATE-seq experiment was performed in collaboration with Columbia University’s Genome Center. Panc-1 and AsPC-1 pancreatic cancer cells were cultured in 96-well tissue culture-treated plates at optimized density, in 100 μl of their optimal media. After 24 h of incubation, the plates were treated with following compounds: RAF inhibitors: sorafenib, dabrafenib, RAF709, PLX8394 and GDC-0879; MEK inhibitors: trametinib, cobimetinib, binimetinib, selumetinib and rafametinib; and ERK inhibitors: SCH772984, ulixertinib, AZD0364 and ravoxertinib (all drugs were obtained from SelleckChem). Each compound was dosed at the concentration at which the cells were 80% viable after 48 h of treatment. After 24 h of treatment, the medium was replaced with 100 ml of FBS supplemented with 10% DMSO and the plates were frozen at −80 °C prior to PLATE-seq. Detailed protocol for preparation of the automated PLATE-seq experiment was described by Bush et al.^64^.

#### Generation of gene set enrichment analysis signature

The PLATE-Seq FASTQ files were pseudoaligned to the GRCh38 human transcriptome and gene expression was quantified using kallisto (version 0.44.0), tximport package^50^ and biomaRt package^65^. The gene expression was quantified as both raw counts (that is, sequencing fragments per genomic locus) and transcripts per million (that is, sequencing fragments per genomic locus normalized for transcript or gene length and sample sequencing depth). Single sample differential gene expression signatures were computed independently for each one of the two cell lines and then integrated in order to derive a consensus MAPK signature. The *z*-score method was used to generate differential gene expression signatures of each drug-treated sample with respect to the DMSO-treated samples.

### Statistics and Reproducibility

The PRISM screen was performed a single time, with 3 technical replicates for each cell line and tested condition, on a total of 796 cell lines.

In all cell line or organoid viability experiments, growth percentage was calculated by normalizing drug-treated values to DMSO control, which was set to 100%. Four-parameter sigmoidal concentration–response model were fitted to the data from at least three biological replicates. Mean ± s.d. was plotted for each tested dilution.

All western blot experiments were run on samples coming from at least three separate biological experiments. Representative images of one experiment are presented in the main figures and extended data figures. In some cases, the same sample sets were run on multiple gels in parallel, with the same loading conditions, to allow probing for proteins of similar molecular weight.

In graphs using box and whisker plots (Fig. 2a,f), the centre line shows the mean, the box boundaries show the 25th and 75th percentile, respectively, and the whiskers show the range of the data.

Statistical analysis (statistical tests and generated *P* values) performed for the data presented in this manuscript is summarized in Supplementary Table 4.

### Reporting summary

Further information on research design is available in the Nature Portfolio Reporting Summary linked to this article.

## Online content

Any methods, additional references, Nature Portfolio reporting summaries, source data, extended data, supplementary information, acknowledgements, peer review information; details of author contributions and competing interests; and statements of data and code availability are available at 10.1038/s41586-024-07379-z.

## Supplementary information

Supplementary Fig. 1 uncropped western blot images with marked areas of interest, and target molecular weight.

Reporting Summary

Supplementary Tables This file contains Supplementary Tables 1–10.

## Source data

Source Data Figs. 1–6

Source Data Extended Data Figs. 1–7

## Data Availability

The entire dataset generated in PRISM cell line multiplex screen has been deposited and reported in the accompanying Article^3^. Global proteomic data have been deposited to the ProteomeXchange Consortium via the PRIDE partner repository. Data are available with the identifiers PXD047878 and PXD047878. The data for spare full genome sequencing is available with Short Read Archive (SRA) accession code PRJNA1083582. Raw data (FASTQs) and processed data (raw counts) for generation of experimental MAPK pathway gene expression signature have been deposited to GEO under accession number GSE252002. Mouse genome build mm9 was used to map sequencing reads from sparse whole-genome sequencing (https://www.ncbi.nlm.nih.gov/datasets/genome/GCF_000001635.18/). GRCh38 human transcriptome was used to pseudoalign human PDAC cell line PLATE-seq sequencing reads (https://www.ncbi.nlm.nih.gov/datasets/genome/GCF_000001405.26/). Experimental data supporting the findings of this study are available within the paper and its Supplementary Information. Information on mouse model, cell line, antibody, primer sequence, reagent, and statistical analysis is provided in the Supplementary Tables 1–10. Source data are provided with this paper.

## Code availability

The code used to analyse PRISM cell line multiplex screen dataset has been deposited and reported in the accompanying Article^3^. For single nucleotide variant calling the full pipeline is available at https://github.com/soccin/BIC-variants_pipeline and the post-processing code is at https://github.com/soccin/Variant-PostProcess. The full source code for sparse whole-genome and targeted locus sequencing methods is available at https://github.com/soccin/seqCNA. An example of the code for the generation of experimental MAPK pathway gene expression signature is available on GitHub at https://github.com/califano-lab/MAPK_Experimental_Signature.git.
