# Author Correction: Tumour-selective activity of RAS-GTP inhibition in pancreatic cancer

**DOI:** 10.1038/s41586-024-08084-7

**Published:** 2024-11-12

**Authors:** Urszula N. Wasko, Jingjing Jiang, Tanner C. Dalton, Alvaro Curiel-Garcia, A. Cole Edwards, Yingyun Wang, Bianca Lee, Margo Orlen, Sha Tian, Clint A. Stalnecker, Kristina Drizyte-Miller, Marie Menard, Julien Dilly, Stephen A. Sastra, Carmine F. Palermo, Marie C. Hasselluhn, Amanda R. Decker-Farrell, Stephanie Chang, Lingyan Jiang, Xing Wei, Yu C. Yang, Ciara Helland, Haley Courtney, Yevgeniy Gindin, Karl Muonio, Ruiping Zhao, Samantha B. Kemp, Cynthia Clendenin, Rina Sor, William P. Vostrejs, Priya S. Hibshman, Amber M. Amparo, Connor Hennessey, Matthew G. Rees, Melissa M. Ronan, Jennifer A. Roth, Jens Brodbeck, Lorenzo Tomassoni, Basil Bakir, Nicholas D. Socci, Laura E. Herring, Natalie K. Barker, Junning Wang, James M. Cleary, Brian M. Wolpin, John A. Chabot, Michael D. Kluger, Gulam A. Manji, Kenneth Y. Tsai, Miroslav Sekulic, Stephen M. Lagana, Andrea Califano, Elsa Quintana, Zhengping Wang, Jacqueline A. M. Smith, Matthew Holderfield, David Wildes, Scott W. Lowe, Michael A. Badgley, Andrew J. Aguirre, Robert H. Vonderheide, Ben Z. Stanger, Timour Baslan, Channing J. Der, Mallika Singh, Kenneth P. Olive

**Affiliations:** 1 Department of Medicine, Vagelos College of Physicians and Surgeons, Columbia University Irving Medical Center, New York, NY USA; 2 Herbert Irving Comprehensive Cancer Center, Columbia University Irving Medical Center, New York, NY USA; 3 Revolution Medicines, Redwood City, CA USA; 4 Department of Cell Biology and Physiology, University of North Carolina at Chapel Hill, Chapel Hill, NC USA; 5 University of Pennsylvania Perelman School of Medicine, Department of Medicine, Philadelphia, PA USA; 6 Cancer Biology and Genetics Program, Sloan Kettering Institute, Memorial Sloan Kettering Cancer Center, New York, NY USA; 7 Lineberger Comprehensive Cancer Center, University of North Carolina at Chapel Hill, Chapel Hill, NC USA; 8 Department of Pharmacology, University of North Carolina at Chapel Hill, Chapel Hill, NC USA; 9 Department of Medical Oncology, Dana-Farber Cancer Institute, Boston, MA USA; 10 Harvard Medical School, Boston, MA USA; 11 University of Pennsylvania Perelman School of Medicine, Abramson Cancer Center, Philadelphia, PA USA; 12 The Broad Institute of Harvard and MIT, Cambridge, MA USA; 13 Department of Systems Biology, Columbia University Irving Medical Center, New York, NY USA; 14 Bioinformatics Core, Memorial Sloan Kettering Cancer Center, New York, NY USA; 15 UNC Michael Hooker Proteomics Center, University of North Carolina at Chapel Hill, Chapel Hill, NC USA; 16 Department of Surgery, Vagelos College of Physicians and Surgeons, Columbia University Irving Medical Center, New York, NY USA; 17 Department of Pathology, H. Lee Moffitt Cancer Center and Research Institute, Tampa, FL USA; 18 Department of Tumor Microenvironment and Metastasis, H. Lee Moffitt Cancer Center and Research Institute, Tampa, FL USA; 19 Department of Pathology and Cell Biology, Columbia University Irving Medical Center, New York, NY USA; 20 Department of Oncology, The Johns Hopkins University School of Medicine, Baltimore, MD USA; 21 J. P. Sulzberger Columbia Genome Center, Columbia University, New York, NY USA; 22 Department of Biochemistry and Molecular Biophysics, Columbia University Irving Medical Center, New York, NY USA; 23 Department of Biomedical Informatics, Columbia University Irving Medical Center, New York, NY USA; 24 Chan Zuckerberg Biohub New York, New York, NY USA; 25 Howard Hughes Medical Institute, Memorial Sloan Kettering Cancer Center, New York, NY USA; 26 Department of Medicine, Brigham and Women’s Hospital, Boston, MA USA; 27 Parker Institute for Cancer Immunotherapy, San Francisco, CA USA; 28 Department of Biomedical Sciences, School of Veterinary Medicine, The University of Pennsylvania, Philadelphia, PA USA

**Keywords:** Pancreatic cancer, Pharmacodynamics

Correction to: *Nature* 10.1038/s41586-024-07379-z Published online 8 April 2024

In the version of the article initially published, in the Data availability section, the GEO accession number was incorrect and has now been amended to GSE252002 in the HTML and PDF version of the article.

