# Supplementary material for: Tumour-selective activity of RAS-GTP inhibition in pancreatic cancer
Source: Nature. 2024 Apr 8;629(8013):927–36. doi: 10.1038/s41586-024-07379-z (PMC11111406; doi:10.1038/s41586-024-07379-z)

nature portfolio

https://doi.org/10.1038/s41586-024-07379-z

Supplementary information

Tumour-selective activity of RAS-GTP inhibition in pancreatic cancer

In the format provided by the authors and unedited

Figure 1e - HPAC

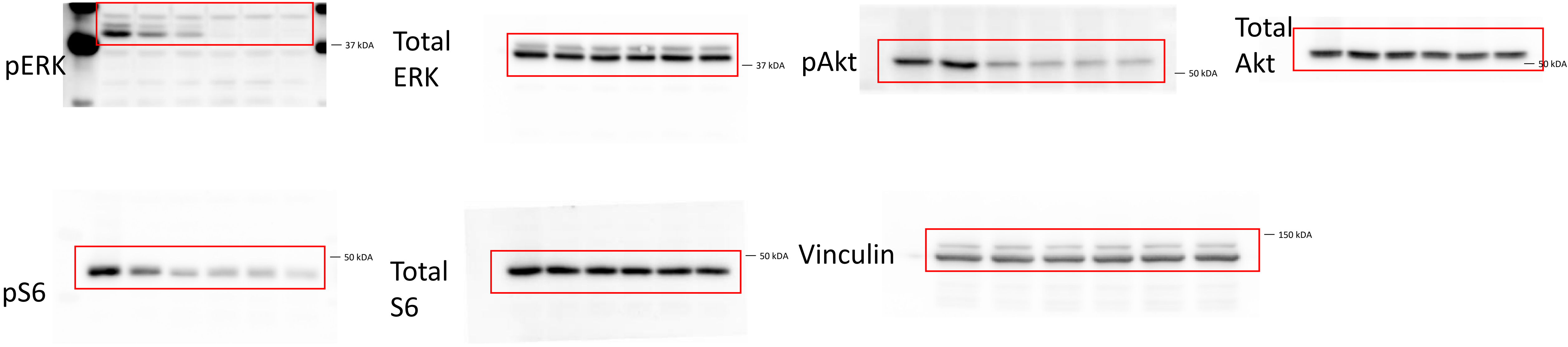

Extended Data Figure 1d – Pa14C

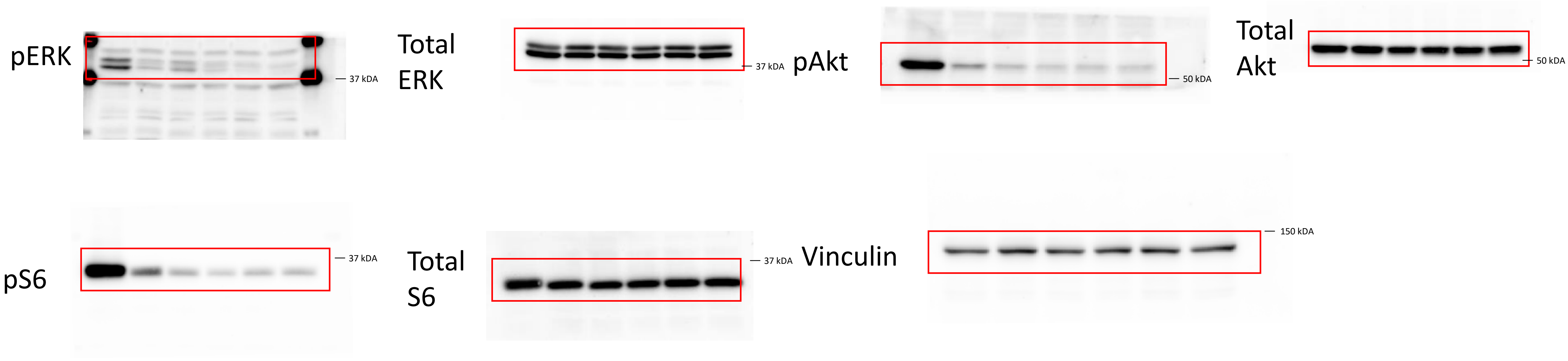

Extended Data Figure 1d – Panc1

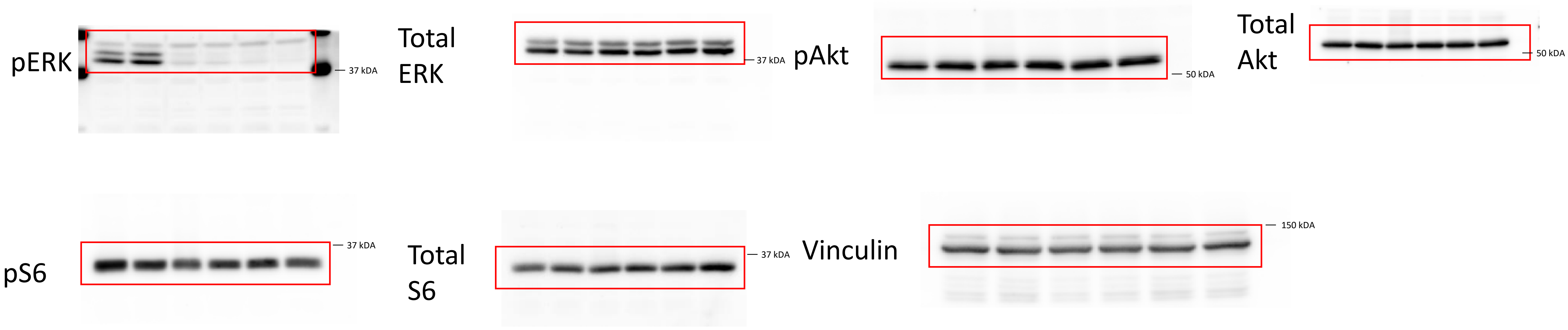

Extended Data Figure 1d – Pa16C

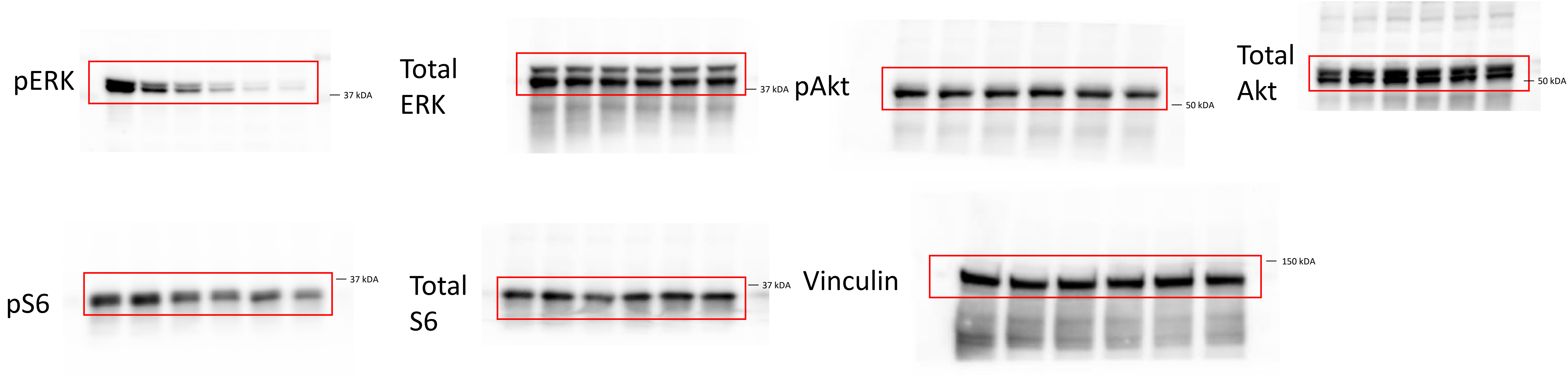

Extended Data Figure 1d – Pa01C

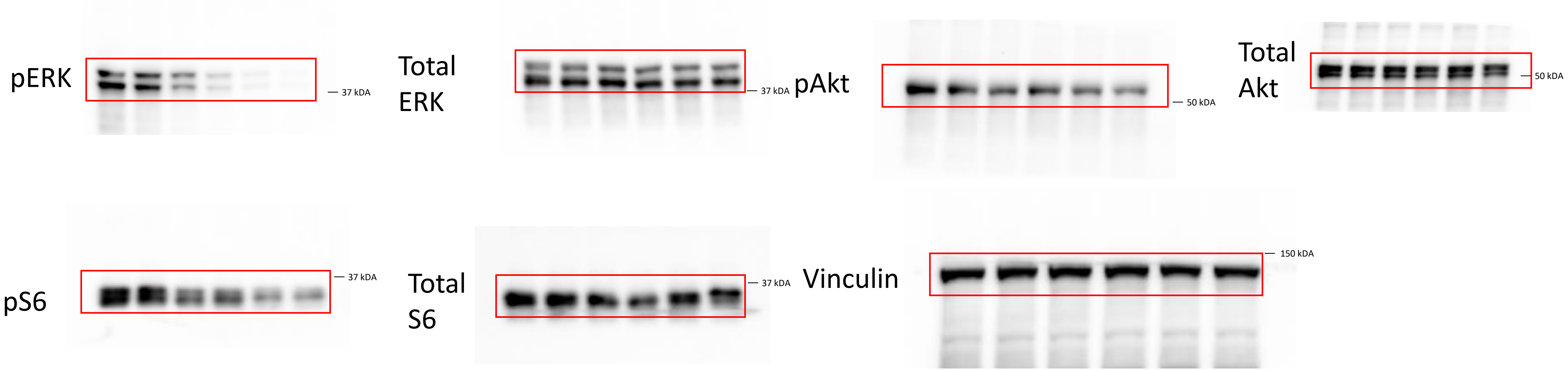

Figure 1f

Extended Data Figure 1e

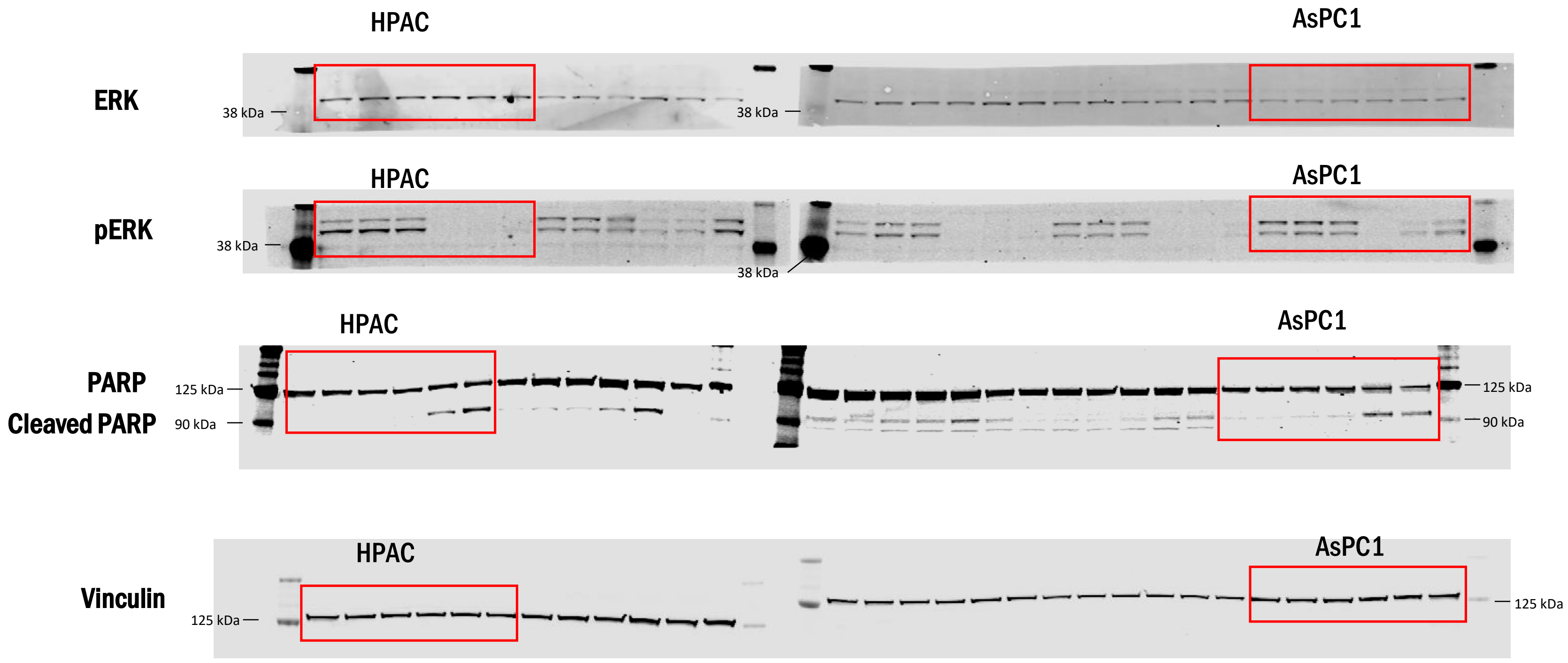

Extended Data Fig 1c

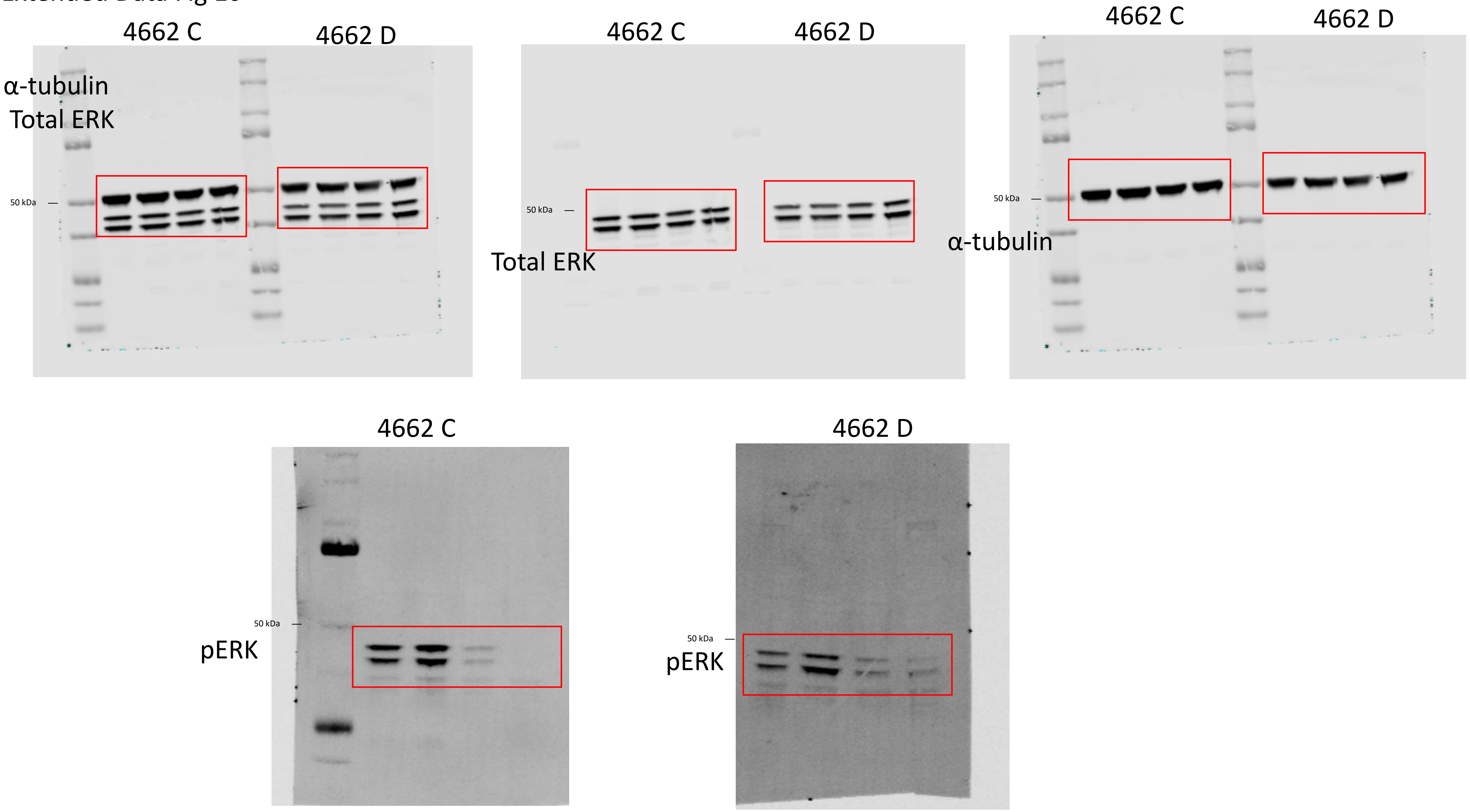

Extended Data Fig 1c

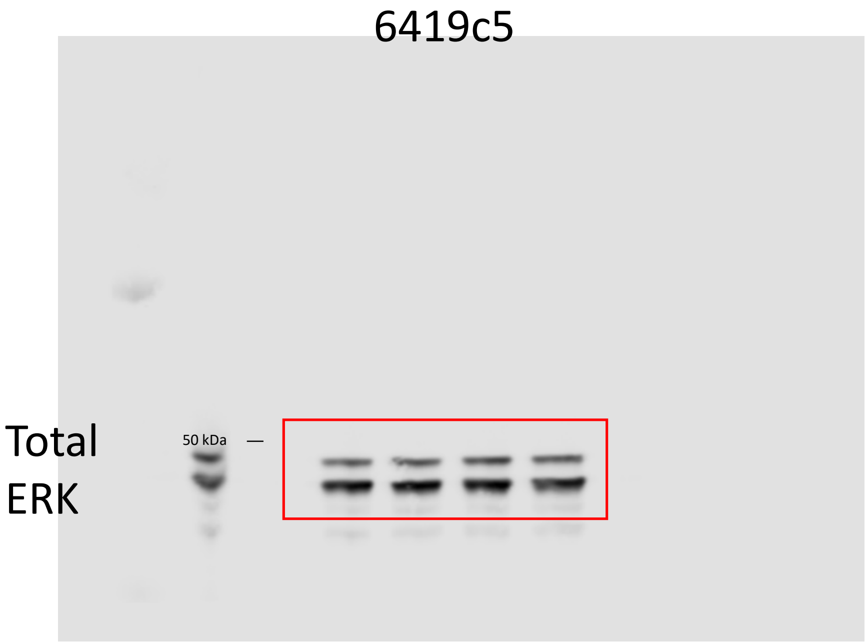

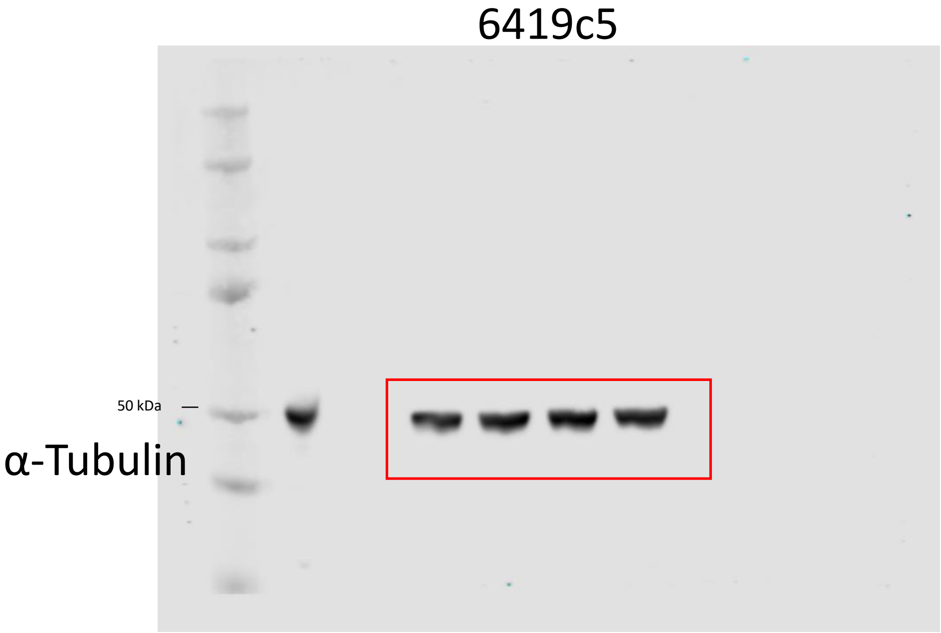

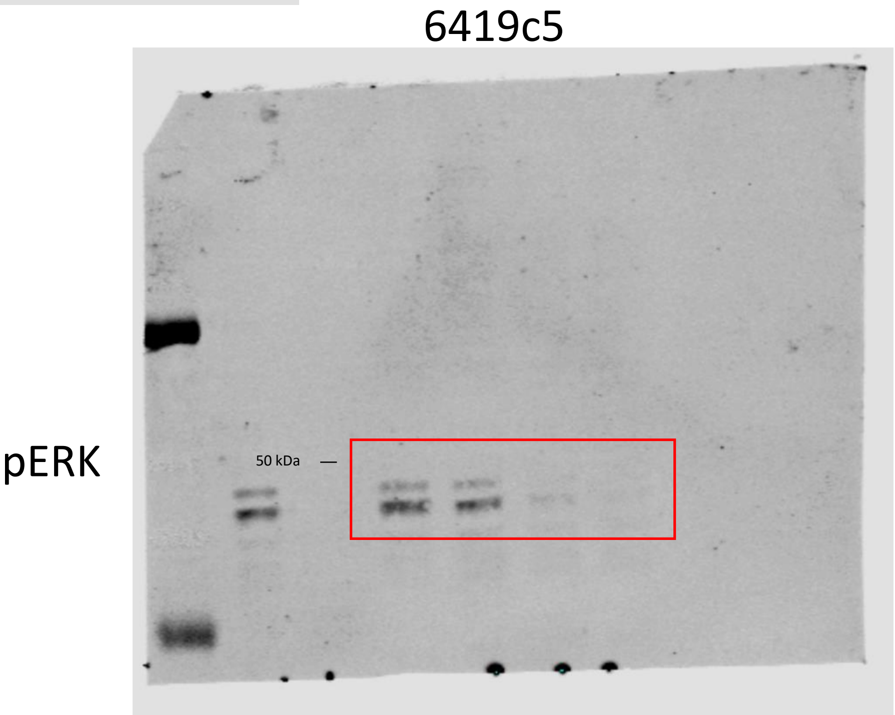

Figure 6f – K2293

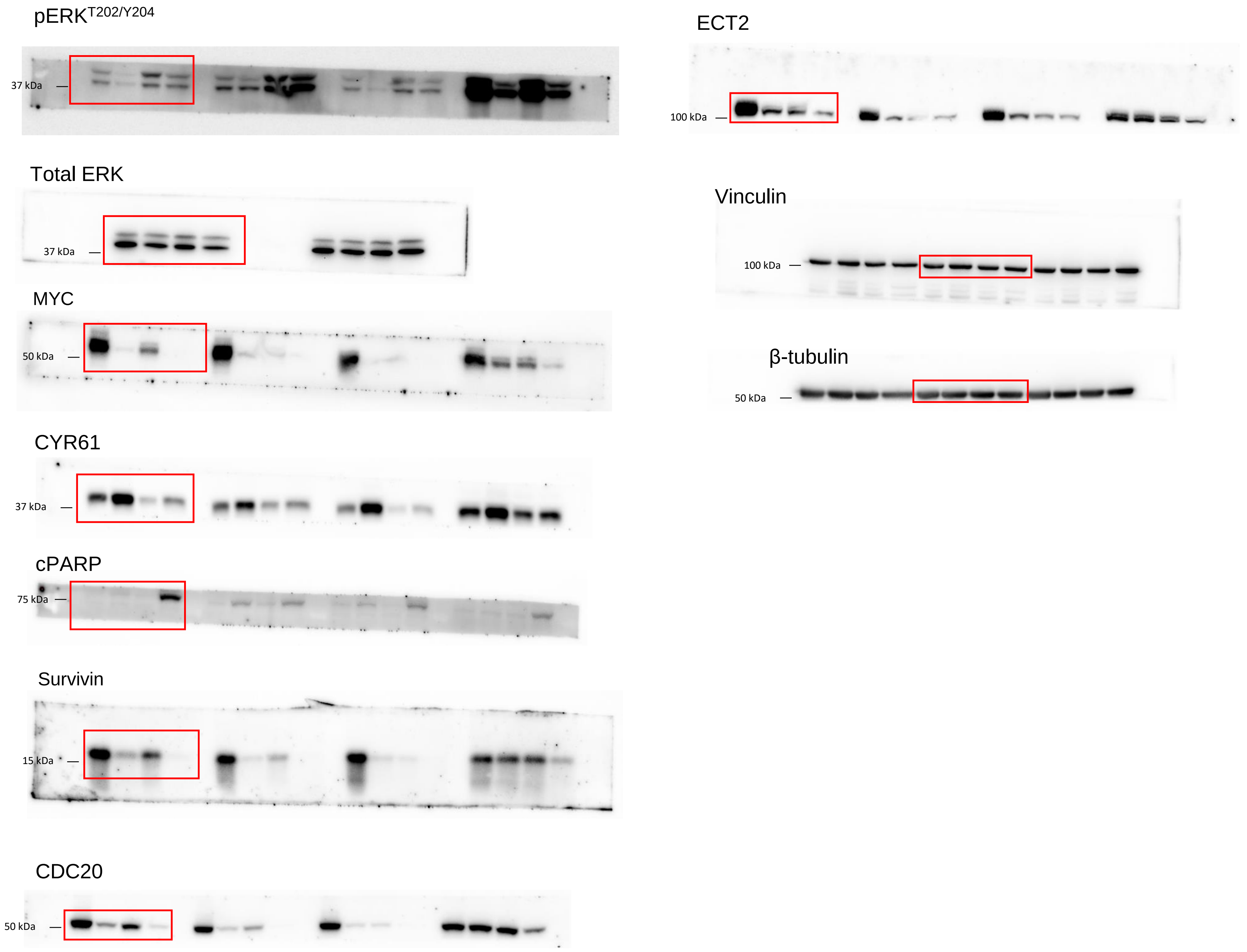

Figure 6f – K8484

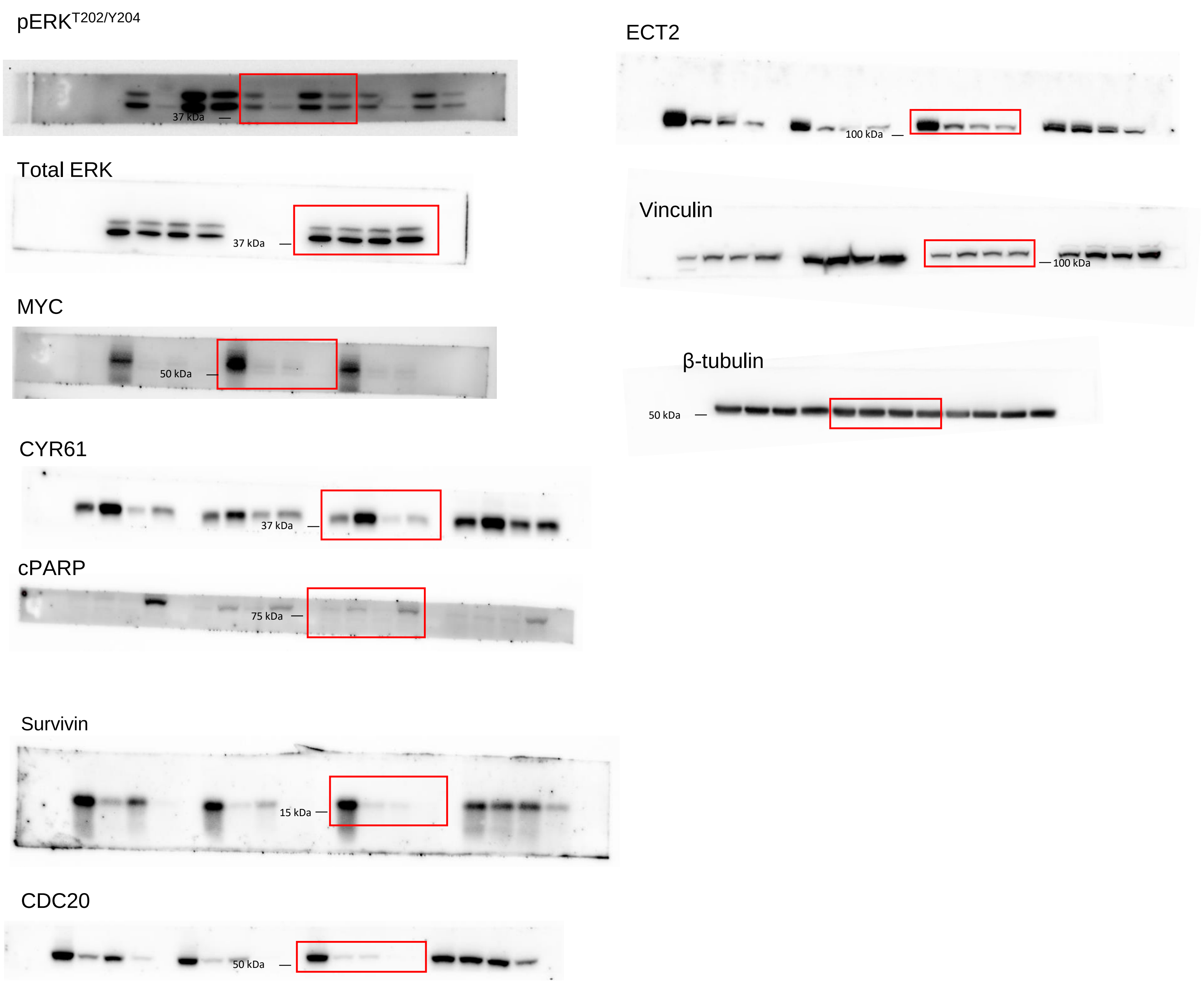

Figure 6f – K18745R

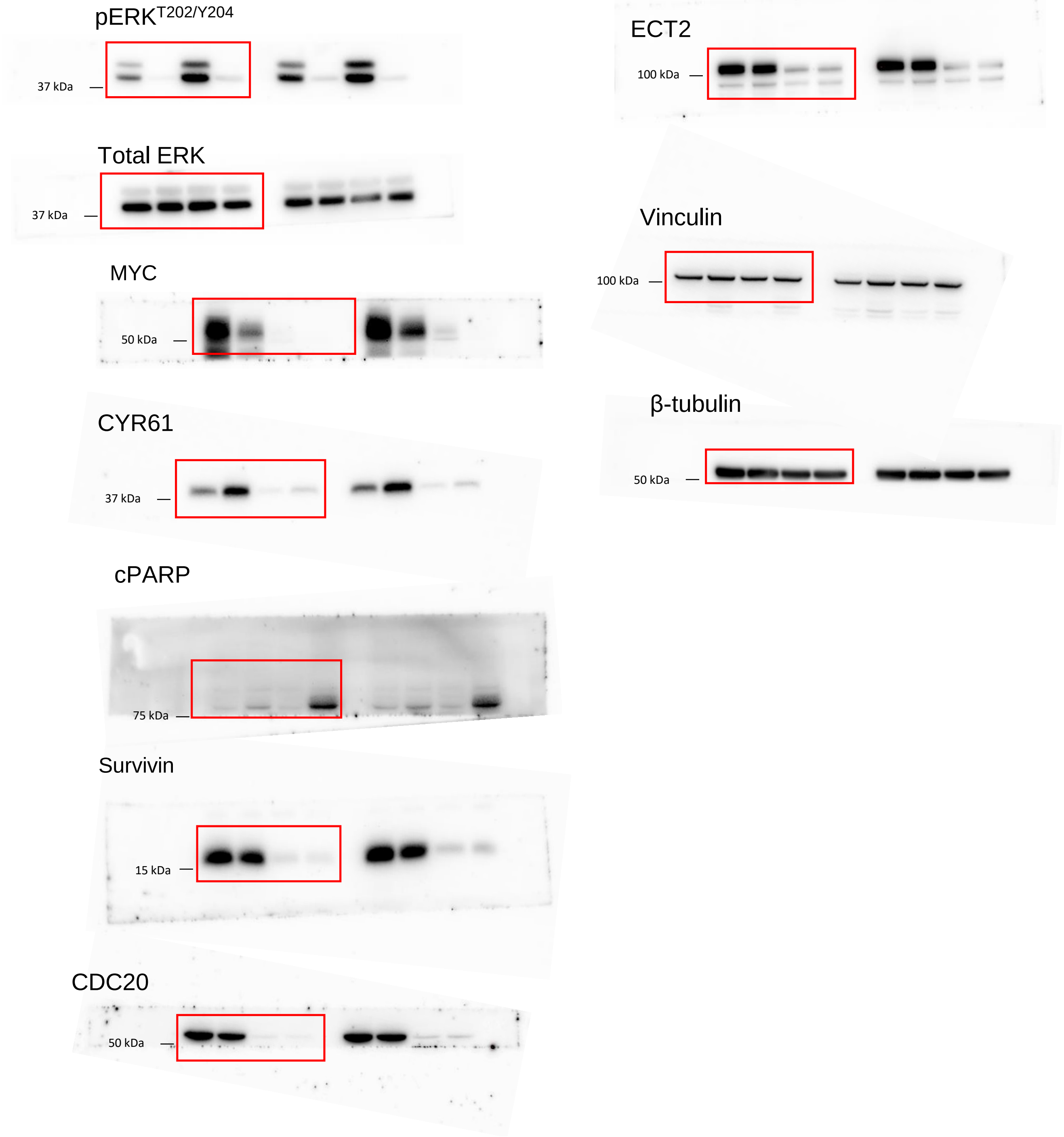

Figure 6f – K18509R

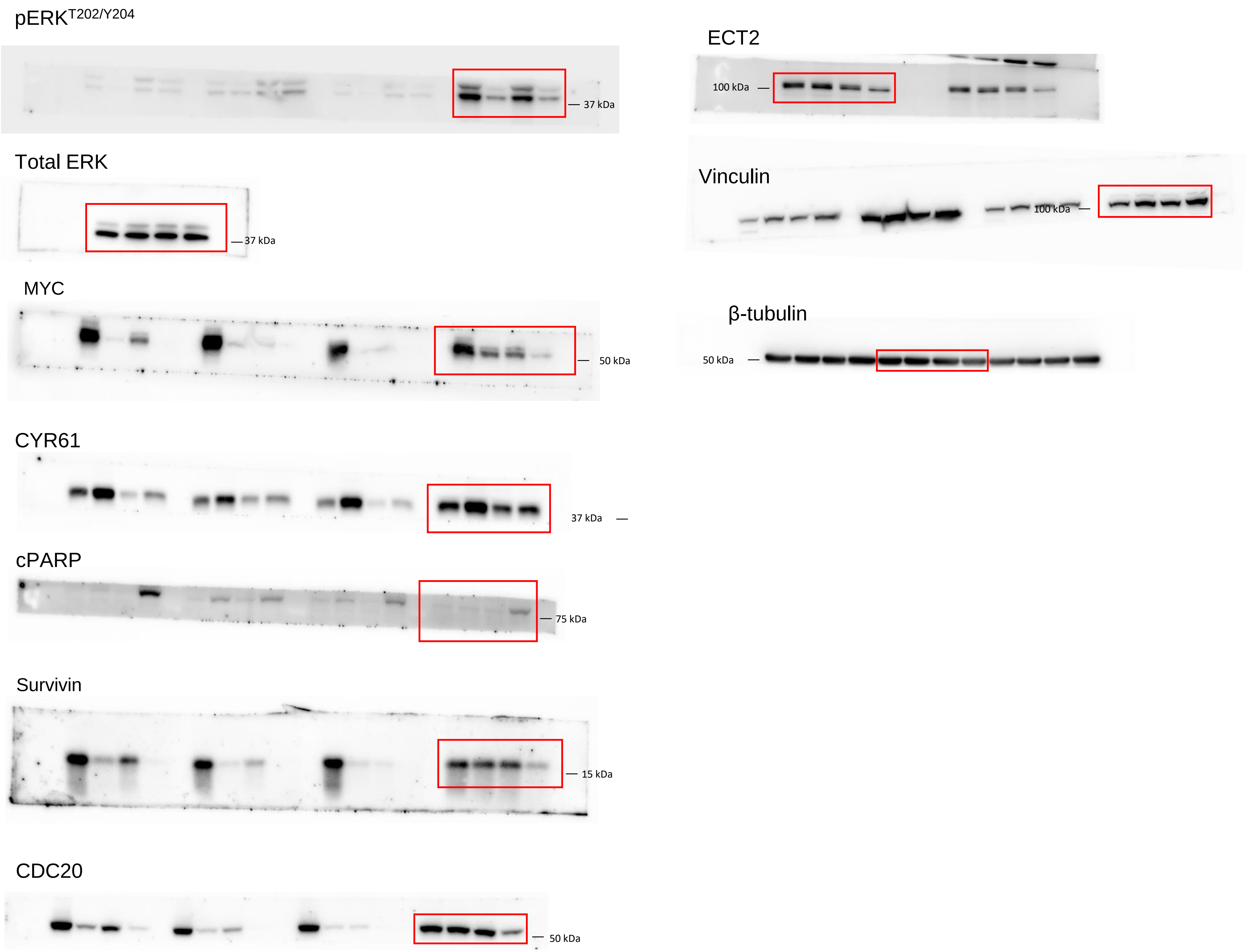

Supplement: Supplementary file 1 — uncropped western blot images with marked areas of interest, and target molecular weight. [file 41586_2024_7379_MOESM1_ESM.pdf]
