# Supplementary material for: Tumour-selective activity of RAS-GTP inhibition in pancreatic cancer
Source: Nature. 2024 Apr 8;629(8013):927–36. doi: 10.1038/s41586-024-07379-z (PMC11111406; doi:10.1038/s41586-024-07379-z)
Supplement: Supplementary file 2 — Reporting Summary [file 41586_2024_7379_MOESM2_ESM.pdf]

nature portfolio

Corresponding author(s): Mallika Singh, Kenneth P. Olive

Last updated by author(s): Mar 16, 2024

Reporting Summary

Nature Portfolio wishes to improve the reproducibility of the work that we publish. This form provides structure for consistency and transparency in reporting. For further information on Nature Portfolio policies, see our Editorial Policies and the Editorial Policy Checklist.

Statistics

For all statistical analyses, confirm that the following items are present in the figure legend, table legend, main text, or Methods section.

n/a	Confirmed	
☐	☒	The exact sample size (*n*) for each experimental group/condition, given as a discrete number and unit of measurement
☐	☒	A statement on whether measurements were taken from distinct samples or whether the same sample was measured repeatedly
☐	☒	The statistical test(s) used AND whether they are one- or two-sided<br>*Only common tests should be described solely by name; describe more complex techniques in the Methods section.*
☐	☒	A description of all covariates tested
☐	☒	A description of any assumptions or corrections, such as tests of normality and adjustment for multiple comparisons
☐	☒	A full description of the statistical parameters including central tendency (e.g. means) or other basic estimates (e.g. regression coefficient) AND variation (e.g. standard deviation) or associated estimates of uncertainty (e.g. confidence intervals)
☐	☒	For null hypothesis testing, the test statistic (e.g. *F*, *t*, *r*) with confidence intervals, effect sizes, degrees of freedom and *P* value noted<br>*Give P values as exact values whenever suitable.*
☒	☐	For Bayesian analysis, information on the choice of priors and Markov chain Monte Carlo settings
☒	☐	For hierarchical and complex designs, identification of the appropriate level for tests and full reporting of outcomes
☒	☐	Estimates of effect sizes (e.g. Cohen's *d*, Pearson's *r*), indicating how they were calculated

Our web collection on statistics for biologists contains articles on many of the points above.

Software and code

Policy information about availability of computer code

Data collection	Software used in PRISM cell line multiplex screen have been reported in the manuscript by Holderfield et al (DOI: 10.21203/rs.3.rs-3122478/v1)<br>LI-COR ImageStudio Lite and ChemiDoc XRS+ or ChemiDocTM MP imager (Bio-Rad) for western blot data collection.<br><br>SpectraMax i3X multimode detection platform and D300e digital dispenser (Tecan) - cell line viability experiments, viability data collection<br><br>Living Image software (Perkin Elmer) - collection of bioluminescence data (tumor volumes)<br><br>Sciex 6500+ triple quadrupole mass spectrometer equipped with an ACQUITY or Shimadzu UPLC system - Pharmacokinetic analysis<br><br>Leica BOND, Biocare IntelliPATH, Leica XL ST5010 - automated staining systems<br><br>TTissueScope LE (Huron Digital Pathology); 3DHistotech Pannoramic whole slide scanner - tissue scanners for histological analysis<br><br>HALO (v3.5.3577.265) software from Indica Labs for Histopathology data collection;<br><br>Vevo 3100 - longitudinal images collection for tumor volume evaluation;<br><br>PALM MicroBeam microscope (Zeiss) - collection of epithelial cells (laser capture microdissection)<br><br>HiSeq Illumina Systems - collection of WG sequencing data

	Thermo Ultimate 3000 nLC coupled to an Exploris480 mass spectrometer (Thermo Scientific) - proteomic data collection
Data analysis	Code availability:<br>1. The code used to analyze PRISM cell line multiplex screen dataset has been deposited and reported in the manuscript by Holderfield et al.<br>2. For single nucleotide variant calling the full pipeline is available here:<br>https://urldefense.com/v3/__https://github.com/soccin/BIC-variants_pipeline__;!!IBzWLUs!Vf0qu3H9amZb4Y0soi9T8UM2id_ByeORtN7wL9E_Q-4jq0VXL2ZYeYAvMcMxYAZcyaIskzioJvsfrG5t8Q$ and the post processing code is at https://urldefense.com/v3/__https://github.com/soccin/Variant-PostProcess__;!!IBzWLUs!Vf0qu3H9amZb4Y0soi9T8UM2id_ByeORtN7wL9E_Q-4jq0VXL2ZYeYAvMcMxYAZcyaIskzioJvuP-V1kqg$<br>3. The full source code for sparse whole genome and targeted locus sequencing methods is available at: https://urldefense.com/v3/__https://github.com/soccin/seqCNA__;!!IBzWLUs!Vf0qu3H9amZb4Y0soi9T8UM2id_ByeORtN7wL9E_Q-4jq0VXL2ZYeYAvMcMxYAZcyaIskzioJvtdDS5nQQ$<br>4. An example of the code for the generation of experimental MAPK pathway gene expression signature is available on GitHub: https://github.com/califano-lab/MAPK_Experimental_Signature.git<br><br>Software used for data analysis:<br>- Synergy Finder v3.10.3 for R(Studio) - for synergy analysis based on cell line viability<br>-Graphpad Prism (v.9.3.1) for statistical analyses and graphical representation<br>-LI-COR ImageStudio Lite for western blot data analysis.<br>-Image analysis and quantification was done using HALO (v3.4, Indica Labs), specifically tissue classifier (v3.5) for tissue class separation; AI mininet module (v3.5) for tissue class separation and; area quantification module (v2.4.2) for quantification of stained tissue. In addition ImageJ-64bit/Fiji was also used for IHC image analysis and quantification<br>-Vevo Lab 5.7.1. and Living Image software (Perkin Elmer) were use to calculate tumor volumes from collected longitudinal images<br>-ImageLab 5.2 was used to aquire and process Western Blot images<br>-Genome sequencing data manipulation and analysis was carried out using: LOWESS smoothing algorithm, Circular Binary Segmentation (CBS), BWA mapper (bwa mem v0.7.12), seqDNAcopy library, cutadapt (v1.6), PICARD tools, GATK toolkit (v 3.2), ABRA (v 0.92), BaseQRecalibrator, muTect (v1.1.7), Haplotype caller.<br>-Generation of the MAPK gene expression signature was carried out using kallisto (version 0.44.0), tximport package (Soneson et al., 2015), and biomaRt package (Durinck et al., 2009).<br>-Proteomic data manipulation and analysis was carried out using: MAXQuant (MQ) 2.4.3.0 Andromeda search engine, R (v4.3.1) using LIMMA (v3.56.2). Gene set enrichment analysis was performed on differential expression analysis results using msigdbr (v7.5.1) and fgsea (v1.26.0).

For manuscripts utilizing custom algorithms or software that are central to the research but not yet described in published literature, software must be made available to editors and reviewers. We strongly encourage code deposition in a community repository (e.g. GitHub). See the Nature Portfolio guidelines for submitting code & software for further information.

Data

Policy information about availability of data

All manuscripts must include a data availability statement. This statement should provide the following information, where applicable:

- Accession codes, unique identifiers, or web links for publicly available datasets
- A description of any restrictions on data availability
- For clinical datasets or third party data, please ensure that the statement adheres to our policy

Data Availability
1. The entire dataset generated in PRISM cell line multiplex screen has been deposited and reported in the companion manuscript by Holderfield et al.
2. Global proteomic data have been deposited to the ProteomeXchange Consortium via the PRIDE partner repository. Data are available with the identifier PXD047878 and 10.6019/PXD047878.
3. The data for spare full genome sequencing is available with Short Read Archive (SRA) accession code: PRJNA1083582.
4. Raw data (FASTQs) and processed data (raw counts) for generation of experimental MAPK pathway gene expression signature have been deposited to GEO, accession number GSE25002.
5. Experimental data supporting the findings of this study are available within the paper and its Supplementary Information. Mouse model, cell line, antibody, primer sequences, reagents, and statistical analysis information are provided in in Supplementary Tables 1-6 and Extended Data Tables 1-4. Raw data for Figures and Extended Figures provided in Excel File format- Raw Data for the Main Figures and Raw Data for the Extended Data Figures. Uncropped Western Blot images are provided as PDF (Supplementary Figure 1)

Mouse genome build mm9 - used to map sequencing reads from sparse whole genome sequencing (https://www.ncbi.nlm.nih.gov/datasets/genome/GCF_000001635.18/)
GRCh38 human transcriptome - used to pseudoalign human PDAC cell line PLATE-Seq sequencing reads (https://www.ncbi.nlm.nih.gov/datasets/genome/GCF_000001405.26/)

Research involving human participants, their data, or biological material

Policy information about studies with human participants or human data. See also policy information about sex, gender (identity/presentation), and sexual orientation and race, ethnicity and racism.

Reporting on sex and gender	Patients were recruited regardless of sex or gender and represent an all-comer cross-section of the patient population served by the Herbert Irving Comprehensive Cancer Center at Columbia University Irving Medical Center (ex vivo human PDAC explants). Sex or gender are not utilized in this manuscript. The justification for this is that the study was not aimed at studying sex or gender differences in pancreatic cancer patients and the overall sample size (N=4 tumor explants) was not sufficient to study additional variables.

Reporting on race, ethnicity, or other socially relevant groupings	Patients were recruited regardless of any racial, ethnic, or social category and represent an all-comer cross-section of the patient population served by the Herbert Irving Comprehensive Cancer Center at Columbia University Irving Medical Center (ex vivo human PDAC explants). Racial, ethnic, or social categories are not utilized in the manuscript.
Population characteristics	All enrolled patients have pathologically-confirmed pancreatic ductal adenocarcinoma that underwent resection at Columbia University Irving Medical Center (ex vivo human PDAC explants), with the following characteristics:<br>Age: 76-85 years<br>Gender: 50% male, 50% female<br>Race/Ethnicity: 50% Hispanic, 50% Unknown<br>Diagnosis: Pancreatic Ductal Adenocarcinoma<br>Prior treatment: 50% - no prior treatment, 50% - unknown
Recruitment	All patients recruited on this study were pancreatic cancer patients who underwent resection surgery at Columbia University Irving Medical Center (ex vivo PDAC explants). Patients were recruited via the Herbert Irving Comprehensive Cancer Center Database Shared Resource or the Columbia University Biobank.
Ethics oversight	Ethical oversight of the human explant experiment was provided by the Columbia University Institutional Review Board, IRB #AAAS6152. Human PDX models were acquired through commercial vendors. For organoid generaton, all patients consented to an Institutional Review Board (IRB)-approved protocol at Dana-Farber Cancer Institute permitting access to their clinical and genomic data.

Note that full information on the approval of the study protocol must also be provided in the manuscript.

Field-specific reporting

Please select the one below that is the best fit for your research. If you are not sure, read the appropriate sections before making your selection.

☒ Life sciences ☐ Behavioural & social sciences ☐ Ecological, evolutionary & environmental sciences

For a reference copy of the document with all sections, see nature.com/documents/nr-reporting-summary-flat.pdf

Life sciences study design

All studies must disclose on these points even when the disclosure is negative.

Sample size	Sample size for the KPC mouse survival study was based on an a priori analysis of statistical power for overall survival of tumor-bearing mice in this model. For all other studies, sample sizes were not predetermined based on statistical methods, but were chosen according to the standards of the field (at least three independent biological replicates for each condition, at least two technical replicates for each in vitro biological assay), which gave sufficient statistics for the effect sizes of interest.
Data exclusions	One tumor (1 week, 24 hrs timepoint) was excluded from the histological analysis of KPC mice in Fig 5 because the tissue fragment available for this analysis was reflected high grade PanINs rather than PDAC. A separate tissue sample from the same mouse and frozen for qRT-PCR analysis, was confirmed to contain PDAC. Normal tissues from this animal were also used for histological analyses. For one KPC mouse in Fig 5 (single dose, 4hr timepoint) skin tissue was not collected at necropsy. Other tissues from this animal were used for analysis. For one KPC mouse in 1 week (4 hr) intervention study, a final ultrasound was not performed and therefore tumor volume analysis was not performed for this animal; tissues from this animal were used for analysis. For one mouse in the KPCY 2-week intervention study, weight measurements were not available. At the cutoff point for data collection, RNA was available from 41 of the 44 KPC mice in Fig 5. and these samples were used for qRT-PCR analysis. One Vehicle treated animal from KPC survival study was found dead and tissue was not collected. Another animal from this study (RMC-7977 arm) was still alive at the time of data cutoff and therefore was not included in any downstream analyses (tumor volume growth, IHC, RT-PCR). No other data were excluded.
Replication	Reported results were consistently replicated across multiple experiments with all replicates generating similar results. Experimental in vitro/ quantitative assays were performed at least in three independent biological replicates and included at least two technical replicates. One exception was the PRISM screen in figure 1a, which was performed one time, with 3 technical replicates per cell line/condition, on 796 cell lines. A second exception was ED Fig 6h, which also performed once, treating each cell line as a separate biological replicate (n=8)). Each of the in vivo study groups included at least 3 animals. IHC image analysis involved quantification of at least 10 independent fields of view. Finally, the main findings of the KPC short-term intervention study (Fig. 5h-k) were independently replicated in the closely-related KPCY model (Fig. 5d-g), performed by different personnel, at a separate institution, using a different genetic background, drug dose, and dosing schedule.
Randomization	In all the in vivo studies presented in the paper, tumor-bearing animals were sequence-randomized into treatment arms. Both male and female mice were used to control for sex as a biological variable.
Blinding	Enrollment of each individual animal on the KPC study was performed in a blinded manner by investagators (SAS and CFP) who did not know the planned treatment for that animal. Once enrolled, treatments were administered without blinding due to personnel limitations.

Reporting for specific materials, systems and methods

We require information from authors about some types of materials, experimental systems and methods used in many studies. Here, indicate whether each material, system or method listed is relevant to your study. If you are not sure if a list item applies to your research, read the appropriate section before selecting a response.

Materials & experimental systems

n/a	Involved in the study
☐	☒ Antibodies
☐	☒ Eukaryotic cell lines
☒	☐ Palaeontology and archaeology
☐	☒ Animals and other organisms
☒	☐ Clinical data
☒	☐ Dual use research of concern
☒	☐ Plants

Methods

n/a	Involved in the study
☒	☐ ChIP-seq
☒	☐ Flow cytometry
☒	☐ MRI-based neuroimaging

Antibodies

Antibodies used

The following primary antibodies were used at 1:1000 dilution for Western Blot analysis: anti-phospho-p44/42 MAPK (ERK1/2) T202/Y204 (no. 9101, no. 4370), anti-p44/42 (ERK1/2) (no. 9107, no. 4695; no. 9102; no. 4696), anti-PARP (no. 9532), anti-phospho-AKT (Ser473) (no. 9271), anti-AKT (40D4) (no. 2920), anti-Akt (Thr308) (244F9) (no. 4056), anti-phospho-S6 (Ser235/236) (no. 2211), anti-S6 (54D2) (no. 2317), anti-c-Myc (D84C12) (no.5605), anti-survivin (no.2808), anti-CDC20 (no. 14866) , anti-CYR61 (no. 39382) and anti-vinculin (no. 13901) from Cell Signaling Technology; anti-vinculin (V9131) from Sigma; anti-ECT2 (no. 07-1364) from Millipore. Anti-alpha-tubulin (no. 3873) from CST and anti-beta-tubulin (no. 66240-1-1g) from Proteintech were used at 1:2000 dilution. Anti-c-PARP (no. 9541) from CST was used at 1:750 dilution. The following secondary antibodies were used according to manufacturer's recommendation: Goat anti-rabbit IR800-conjugated (no. 926-32211), goat anti-mouse IR680-conjugated (no. 926-68070), goat anti-mouse IR800-conjugated (no. 926-32210) from Li-COR; HRP-linked anti-rabbit (no. 7074) and HRP-linked anti-mouse (no. 7076) from CST; IgG (H+L) Cross-Adsorbed Goat anti-Mouse, HRP (no. PI31432) and IgG (H+L) Cross-Adsorbed Goat anti-Rabbit, HRP (no. PI31462) from Invitrogen, Goat anti-Mouse IgG (H+L) Cross-Adsorbed Secondary Antibody, HRP (no. 31432), Goat anti-Rabbit IgG (H+L) Cross-Adsorbed Secondary Antibody, HRP (no. 31462) from Thermo Fiisher Scientific.

The primary antibodies used for histology were: anti-phospho-p44/42 MAPK (ERK1/2) T202/Y204 (no. 4370, 1:200 (GEMM and Allograft Models) or 1:1000 (Xenograft Models)), anti- cleaved caspase-3 (Asp175) (no. 9661, 1:200 (GEMM models) or 1:400 (Xenograft Models)), anti-cleaved PARP (no. 94885; 1:200 for GEMM models) and anti-survivin (no. 2808; 1:500 for GEMM models) from Cell Signaling Technology; anti- cleaved caspase-3 (no. CP229, 1:100 (Allograft Models)) from Biocare Medical, anti-cyclin A2 (no. 181591, 1:500 (GEMM models)) from Abcam, anti-phospho-S6 Ribosomal Protein (Ser235/236) (no. 2211, 1:200 (GEMM models) from CST, anti-phospho-S6 Ribosomal Protein (Ser240/244) (D68F8) XP® (no.5364, 1:2000) from CST. Primary antibodies were detected using: ImmPRESS HRP Horse Anti-Rabbit IgG Polymer Detection Kit (no. MP-7401) from Vector Laboratories, MACH4 HRP-polymer Detection System (no. M4U534) from Biocare Medical, and Leica BOND Polymer detection kit (no. 3-P-PV6119) from Leica Microsystems.

The primary antibodies used for IF were: anti-keratin 19 (D7F7W, no 13092, 1:200 dilution), anti-phospho-p44/42 MAPK (ERK1/2) T202/Y204 (no. 4370, 1:100 dilution), anti-phospho-S6 Ribosomal Protein (Ser235/236) (D57.2.2E, no. 4858, 1:100 dilution) from Cell Signalling Technology.

Validation

Antibodies used in the manuscript are well-published commercially available reagents. Validation statements can be found on manufacturers' website:
- anti-phospho-p44/42 MAPK (ERK1/2) T202/Y204 (no. 9101, no. 4370)
https://www.cellsignal.com/products/primary-antibodies/phospho-p44-42-mapk-erk1-2-thr202-tyr204-antibody/9101
https://www.cellsignal.com/products/primary-antibodies/phospho-p44-42-mapk-erk1-2-thr202-tyr204-d13-14-4e-xp-174-rabbit-mab/4370
-anti-p44/42 (ERK1/2) (no. 9107, no. 4695; no. 9102; no. 4696)
https://www.cellsignal.com/products/primary-antibodies/p44-42-mapk-erk1-2-3a7-mouse-mab/9107
https://www.cellsignal.com/products/primary-antibodies/p44-42-mapk-erk1-2-137f5-rabbit-mab/4695
https://www.cellsignal.com/products/primary-antibodies/p44-42-mapk-erk1-2-antibody/9102
https://www.cellsignal.com/products/primary-antibodies/p44-42-mapk-erk1-2-l34f12-mouse-mab/4696
-anti-PARP (no. 9532)
https://www.cellsignal.com/products/primary-antibodies/parp-46d11-rabbit-mab/9532
-anti-phospho-AKT (Ser473) (no. 9271)
https://www.cellsignal.com/products/primary-antibodies/phospho-akt-ser473-antibody/9271
-anti-AKT (40D4) (no. 2920)
https://www.cellsignal.com/products/primary-antibodies/akt-pan-40d4-mouse-mab/2920
-anti-Akt (Thr308) (244F9) (no. 4056)
https://www.cellsignal.com/products/primary-antibodies/phospho-akt-thr308-244f9-rabbit-mab/4056
-anti-phospho-S6 (Ser235/236) (no. 2211)
https://www.cellsignal.com/products/primary-antibodies/phospho-s6-ribosomal-protein-ser235-236-antibody/2211
-anti-S6 (54D2) (no. 2317)
https://www.cellsignal.com/products/primary-antibodies/s6-ribosomal-protein-54d2-mouse-mab/2317
-anti-c-Myc (D84C12) (no.5605)
https://www.cellsignal.com/products/primary-antibodies/c-myc-d84c12-rabbit-mab/5605
-anti-survivin (no.2808)
https://www.cellsignal.com/products/primary-antibodies/survivin-71g4b7-rabbit-mab/2808

-anti-CDC20 (no. 14866)
https://www.cellsignal.com/products/primary-antibodies/cdc20-d6c2q-rabbit-mab/14866
-anti-CYR61 (no. 39382)
https://www.cellsignal.com/products/primary-antibodies/cyr61-e5w3h-rabbit-mab/39382
-anti-vinculin (no. 13901)
https://www.cellsignal.com/products/primary-antibodies/vinculin-e1e9v-xp-174-rabbit-mab/13901
-anti-vinculin (V9131)
https://www.sigmaaldrich.com/US/en/product/sigma/v9131?utm_source=google&utm_medium=cpc&utm_campaign=8890193254&utm_content=87903050565&gclid=CjwKCAjw48-vBhBbEiwAzqrZVHb3dbQZNehAIlON754ph2PjWjlaXuM0TvcIl36zp6ew0aoAlgPcmxoCcREQAvD_BwE
-anti-ECT2 (no. 07-1364)
https://www.emdmillipore.com/US/en/product/Anti-ECT2-Antibody,MM_NF-07-1364?ReferrerURL=https%3A%2F%2Fwww.google.com%2F
-anti-alpha-tubulin (no. 3873)
https://www.cellsignal.com/products/primary-antibodies/a-tubulin-dm1a-mouse-mab/3873
-anti-beta-tubulin (no. 66240-1-1g)
https://www.ptglab.com/products/beta-Tubulin-Antibody-CL488-66240.htm
-anti-c-PARP (no. 9541)
https://www.cellsignal.com/products/primary-antibodies/cleaved-parp-asp214-antibody/9541
-goat anti-rabbit IR800-conjugated (no. 926-32211)
https://www.licor.com/bio/support/contents/reagents/irdye-secondary-antibodies/800cw/goat-anti-rabbit-igg.html
-goat anti-mouse IR680-conjugated (no. 926-68070)
https://www.licor.com/bio/reagents/irdye-680rd-goat-anti-mouse-igg-secondary-antibody
-goat anti-mouse IR800-conjugated (no. 926-32210)
https://www.licor.com/bio/reagents/irdye-800cw-goat-anti-mouse-igg-secondary-antibody
-HRP-linked anti-rabbit (no. 7074)
https://www.cellsignal.com/products/secondary-antibodies/anti-rabbit-igg-hrp-linked-antibody/7074
-HRP-linked anti-mouse (no. 7076)
https://www.cellsignal.com/products/secondary-antibodies/anti-mouse-igg-hrp-linked-antibody/7076
-IgG (H+L) Cross-Adsorbed Goat anti-Mouse HRP (no. PI31432)
https://preview.fishersci.com/shop/products/goat-anti-mouse-igg-h-l-cross-adsorbed-secondary-antibody-hrp-invitrogen/PI31432
IgG (H+L) Cross-Adsorbed Goat anti-Rabbit HRP (no. PI31462)
https://www.fishersci.com/shop/products/goat-anti-rabbit-igg-h-l-hrp-polyclonal-thermo-scientific-pierce-1-5ml-hrp/pi31462
-anti-Mouse IgG (H+L) Cross-Adsorbed Secondary Antibody, HRP (no. 31432)
https://www.thermofisher.com/antibody/product/Goat-anti-Mouse-IgG-H-L-Cross-Adsorbed-Secondary-Antibody-Polyclonal/31432
-goat anti-Rabbit IgG (H+L) Cross-Adsorbed Secondary Antibody, HRP (no. 31462)
https://www.thermofisher.com/antibody/product/Goat-anti-Rabbit-IgG-H-L-Cross-Adsorbed-Secondary-Antibody-Polyclonal/31462
-anti- cleaved caspase-3 (Asp175) (no. 9661)
https://www.cellsignal.com/products/primary-antibodies/cleaved-caspase-3-asp175-antibody/9661
-anti-cleaved PARP (no. 94885)
https://www.cellsignal.com/products/primary-antibodies/cleaved-parp-asp214-d6x6x-rabbit-mab/94885
-anti-survivin (no. 2808)
https://www.cellsignal.com/products/primary-antibodies/survivin-71g4b7-rabbit-mab/2808
-anti- cleaved caspase-3 (no. CP229)
https://biocare.net/product/caspase-3-antibody/
-anti-cyclin A2 (no. 181591)
https://www.abcam.com/products/primary-antibodies/cyclin-a2-antibody-epr17351-ab181591.html
-anti-phospho-S6 Ribosomal Protein (Ser240/244) (D68F8) XP® (no.5364)
https://www.cellsignal.com/products/primary-antibodies/phospho-s6-ribosomal-protein-ser240-244-d68f8-xp-174-rabbit-mab/5364
-ImmPRESS HRP Horse Anti-Rabbit IgG Polymer Detection Kit (no. MP-7401)
https://vectorlabs.com/products/immpress-hrp-horse-anti-rabbit-igg
-MACH4 HRP-polymer (no. M4U534)
https://biocare.net/product/mach-4-universal-hrp-polymer/
-Leica BOND Polymer detection kit (no. 3-P-PV6119)
https://shop.leicabiosystems.com/us/ihc-ish/detection-systems/pid-powervision-poly-hrp-anti-rabbit-ihc-detection-systems
-anti-keratin 19 (D7F7W, no 13092)
https://www.cellsignal.com/products/primary-antibodies/keratin-19-d7f7w-rabbit-mab/13092
-Anti-phospho-S6 Ribosomal Protein (Ser235/236) (D57.2.2E, no. 4858)
https://www.cellsignal.com/products/primary-antibodies/phospho-s6-ribosomal-protein-ser235-236-d57-2-2e-xp-174-rabbit-mab/4858

Eukaryotic cell lines

Policy information about cell lines and Sex and Gender in Research

Cell line source(s)

The following human cell lines were used in the study: AsPC-1, Capan-1, HPAF-II, MIA PaCa-2, Hs 766T and HPAC from ATCC; T3M-4 from Riken; KPC/Y 6499c4 from Dr. Ben Stanger and Dr. Robert Vonderheide (University of Pennsylvania); Pa01C, Pa02C, Pa14C, Pa16C, Panc1, and HPAC from Dr. Anirban Maitra (MD Anderson Cancer Center) and Dr. Channing Der (University of North Carolina); hF39, hF43 from Dr. David Tuveson (CSHL); PaCaDD-137 and PaCaDD-165 obtained from DSMZ; UM147 obtained from University of Michigan (PMID: 17283135); AsPC-1, HPAC, HPAF-II, Panc 05.04, PANC-1, Panc 10.05, PL-45, SU.86.86, KP4, SW1990, PSN1, Capan-1, Capan-2, CFPAC-1, Hup-T4, Panc -3.27, Hs 766T, MIA PaCa-2, and BxPC-3 tested at Crown Bioscience (cell lines sources include ATCC, RIKEN, SIBS, CoBioer, and PUMC).

Tumor fragment based xenograft moldels (PAN022, PAN035, PAN047) were generated from human tissue samples by Genedesign, Inc.

	2838c3 and 6419c5 cell lines were isolated from late-stage primary tumors from female KPCY (KrasLSL-G12D/+;Trp53L/+;Pdx1-Cre;Rosa26YFP/YFP and KrasLSL-G12D/+;Trp53LSL-R172H/+;Pdx1-Cre;Rosa26YFP/YFP) with C57BL/6 background and generated by limiting dilution (Li... B.Z. Stanger, Immunity, 2018)<br>4662 G12D was isolated from female KPCY mice. The 4662-G12C line was generated using CRISPR/Cas9 to replace the endogenous G12D mutation from 4662-G12D cells and with the G12C mutation using lentiviral transduction. KRAS allele states was confirmed by genomic sequencing. (Vonderheide, Science, 2011)<br><br>K2293, K8484 were isolated from late stage primary tumors from female KPC mice by Dr Kenneth Olive. K18509R, K18745R, K18399R, K18850R, K18915R, K18849R were isolated from late stage tumors from KPC mice that were treated with RMC-7977 until endpoint.<br><br>The PRISM cell set consisted of 796 cell lines representing more than 45 lineages (See Supplementary Methods Table 2 for cell line information), which largely overlapped with the Cancer Cell Line Encyclopedia (CCLE) (https://portals.broadinstitute.org/ccle).<br><br>KPCY Naive cell lines and KPF/+C naive tumors sequencing data was provided by Dr Timour Baslan (UPenn).
Authentication	All human cell lines tested were authenticated by STR analysis.<br>Murine cell line Kras allele status (G12D, G12C) was confirmed by genomic sequencing.
Mycoplasma contamination	Cell lines were confirmed negative for mycoplasma through routine testing using the MycoAlert Mycoplasma Detection Kit. (Lonza, LT07-318)
Commonly misidentified lines<br>(See ICLAC register)	No commonly misidentified lines were used.

Animals and other research organisms

Policy information about studies involving animals; ARRIVE guidelines recommended for reporting animal research, and Sex and Gender in Research

Laboratory animals	Mus musculus, Kras LSL-G12D/+; Trp53 LSL-R172H/+, Pdx1-Cre, Rosa26 YFP/YFP (KPCY) on C57BL/6 background, age various<br>Mus musculus C57Bl/6J 6-8 weeks old, BALB/c Nude mouse, NOD SCID mouse<br>Mus musculus: KPC (KrasLSL.G12D/+; p53LSL.R172H/+; Pdx1-Cre), KC (KrasLSL.G12D/+; Pdx1-Cre), PC (p53LSL.R172H/+; Pdx1-Cre) as well as KPF/FC (KrasLSL.G12D/+; p53flox/flox; Pdx1-Cre), KPF/F (KrasLSL.G12D/+; p53flox/flox) and PF/FC (p53flox/flox; Pdx1-Cre) on 129S4/SvJae background<br>Animals were maintained in a 12-hour light cycle, at 70-72 *F, with 35% humidity.
Wild animals	No wild animals were used in the study.
Reporting on sex	Both male and female mice were used to control for sex as a biological variable.
Field-collected samples	No field-collected samples were utilized in this study.
Ethics oversight	All animal experiments and procedures were performed under approved protocols in accordance with guidelines Institutional Animal Care and Use Committees (IACUC) of Columbia University Irving Medical Center, the University of Pennsylvania, Revolution Medicines or associated CROs.

Note that full information on the approval of the study protocol must also be provided in the manuscript.
